# Elevated Water CO_2_ Can Prevent Dietary-Induced Osteomalacia in Post-Smolt Atlantic Salmon (*Salmo salar*, L.)

**DOI:** 10.3390/biom13040663

**Published:** 2023-04-10

**Authors:** Lucia Drábiková, Per Gunnar Fjelldal, Muhammad Naveed Yousaf, Thea Morken, Adelbert De Clercq, Charles McGurk, Paul Eckhard Witten

**Affiliations:** 1 Evolutionary Developmental Biology, Biology Department, Ghent University, Ledeganckstraat 35, 9000 Ghent, Belgium; 2 Institute of Marine Research (IMR), Matre Research Station, N-5984 Matredal, Norway; 3 Skretting Aquaculture Innovation, Sjøhagen 3, 4016 Stavanger, Norway

**Keywords:** CO_2_, dietary phosphorus, bone mineralization, Atlantic salmon, skeleton, *fgf23*

## Abstract

Expansion of land-based systems in fish farms elevate the content of metabolic carbon dioxide (CO_2_) in the water. High CO_2_ is suggested to increase the bone mineral content in Atlantic salmon (*Salmo salar*, L.). Conversely, low dietary phosphorus (P) halts bone mineralization. This study examines if high CO_2_ can counteract reduced bone mineralization imposed by low dietary P intake. Atlantic salmon post-seawater transfer (initial weight 207.03 g) were fed diets containing 6.3 g/kg (0.5P), 9.0 g/kg (1P), or 26.8 g/kg (3P) total P for 13 weeks. Atlantic salmon from all dietary P groups were reared in seawater which was not injected with CO_2_ and contained a regular CO_2_ level (5 mg/L) or in seawater with injected CO_2_ thus raising the level to 20 mg/L. Atlantic salmon were analyzed for blood chemistry, bone mineral content, vertebral centra deformities, mechanical properties, bone matrix alterations, expression of bone mineralization, and P metabolism-related genes. High CO_2_ and high P reduced Atlantic salmon growth and feed intake. High CO_2_ increased bone mineralization when dietary P was low. Atlantic salmon fed with a low P diet downregulated the *fgf23* expression in bone cells indicating an increased renal phosphate reabsorption. The current results suggest that reduced dietary P could be sufficient to maintain bone mineralization under conditions of elevated CO_2_. This opens up a possibility for lowering the dietary P content under certain farming conditions.

## 1. Introduction

The use of increased stocking densities and land-based fish farming systems is inherently associated with the increased accumulation of metabolic carbon dioxide (CO_2_) in the water [1,2]. The maximum, recommended level of dissolved CO_2_ for Atlantic salmon ranges between 8 and 15 mg/L while 20 mg/L is also considered safe by some authors [3,4,5,6,7,8,9,10]. The CO_2_ level has been reported to reach up to 40 mg/L on land-based farms [4]. 

Dissolved CO_2_, carbonate ion (CO_3_^2−^), and bicarbonate ion (HCO_3_^−^) make up the total dissolved inorganic carbon in water. Hydrolyzed carbon dioxide forms H_2_CO_3_, a weak acid that is promptly dissociated into HCO_3_^−^ and a hydrogen ion. Bicarbonate ions bind with free calcium (Ca^2+^) to form CaCO_3_ in the process of calcification [11] whereas the hydrogen ion bonds with CO_3_^2−^. Thus, carbonate is the limiting factor to buffer the increase in CO_2_ [12]. Following reduction in water pH, the environment becomes undersaturated with CO_3_^2−^. Reduced water pH leads to an increased solubility of carbonated minerals such as calcium carbonate (CaCO_3_) [13]. Particulate CaCO_3_ dissolves into Ca^2+^ and CO_3_^2−^ [14]. The buffering capacity of water is dependent on the availability of CO_3_^2−^ and increases with a higher water alkalinity. As seawater has a higher alkalinity compared with freshwater it is a more efficient pH buffer [15,16].

Teleosts represent the most diverse vertebrate group with the ability to adapt to different and changing environmental conditions [17]. The concentration of O_2_ in water is about 14 mg/L and decreases with increasing temperature [18]. Due to reduced water mixing along the coasts and coastal eutrophication, even marine teleost fish can experience transient periods of low O_2_ in the wild. Fish schooling also creates a low oxygen zone inside and especially at the end of the school for individuals located behind other individuals [19]. Teleosts possess gill receptors to sense a reduced O_2_ level. Among other important adaptations is the Root effect, a pH-reducing physiological response of teleost fish which lowers the hemoglobin–O_2_ affinity and enhances O_2_ offloading in tissues with an increased O_2_ requirement [20]. To ensure an efficient O_2_ uptake through gills at times of reduced blood pH, teleost fish produce stress hormones to trigger a sodium/hydrogen proton (Na^+^/H^+^) exchange at the membrane of red blood cells (RBC). The removal of H^+^ increases the pH in RBC and stimulates O_2_ loading at the gills during systemic acidosis associated with low O_2_ uptake and increased blood CO_2_ during exhaustive exercise, such as upstream migration and predator–prey interaction. 

Although teleost fishes can effectively cope with high CO_2_ (hypercapnia), studies show that freshwater and seawater Atlantic salmon (*Salmo salar*, L.) exposed to high CO_2_ concentrations suffer reduced growth and a reduced feed–conversion ratio [4,8,9,21,22,23,24,25]. High water CO_2_ increases plasma pH [9,23,26] as a compensatory response of the organism to the systemic acidosis. Plasma chloride (Cl^−^) is reduced [8,22,23] as a result of the increased Ca^2+^ influx which is required to bind HCO_3_^−^ formed under high ambient CO_2_ [27]. Moreover, hypercapnia has been previously suggested as a factor inducing skeletal deformities [28]. 

The main mineral components of bone are calcium (Ca) and phosphorus (P). As teleosts can supplement sufficient levels of Ca through gill uptake they are, to a large extent, independent from intestinal Ca absorption [29]. Studies on European catfish (*Silurus glanis*), redbelly tilapia (*Coptodon zilli*, Gervais, 1848), and turbot (*Psetta maxima*) suggest that aquatic vertebrates are also able to absorb waterborne P [30,31,32]. The concentration of P in water is however limited and the requirements of P are met predominantly through the diet [33]. 

Commercial diets commonly contain surplus of dietary P with the aim of enhancing bone mineralization, a preventive measure against the development of vertebral deformities [34,35,36,37]. However, dietary P that exceeds the nutritional requirements neither improves bone health nor does it enhance the bone formation or bone mineralization in Atlantic salmon [38,39,40,41]. In addition, excess dietary P leads to vertebral fusions in zebrafish (*Danio rerio*) [42].

Under experimental conditions, a period of low dietary P halts bone mineralization while bone matrix formation is maintained. Parr and the early-seawater stages of Atlantic salmon thus develop bone void of minerals at the edges of vertebral bodies and bone trabeculae (osteomalacia) which leads to reduced vertebral centra stiffness [39,40,43]. Recovery from low-P diet-associated osteomalacia is possible with a diet containing the required level of P [38,40]. 

While low dietary P leads to a reduced bone mineral content and formation of extended areas of non-mineralized bone, high CO_2_ was shown to enhance bone mineralization in farmed teleosts and rodents [1,4,9,44,45]. Gil-Martens et al. [4] showed an increased bone ash content, increased P content, a higher trabecular volume, and increased bone mineralization in Atlantic salmon smolts reared in 35 mg/L CO_2_ for 19 weeks. Similarly, Elsadin et al. [45] reported an increased bone Ca content in white grouper (*Epinephelus aeneus*) reared in 28.5 mg/L CO_2_ for 60 days. The authors proposed an increased water Ca gill uptake and increased intestinal Ca and P absorption as an explanation for the increased Ca bone content. Helland et al. [1] applied a high stocking density in a study on Atlantic salmon parr for 30 days which resulted in a high water CO_2_ (20–25 mg/L) and led to an increased ash, Ca, and P vertebral mineral content. A freshwater history of 24 mg/L CO_2_ lasting for 60 days induced an increased whole body ash, Ca, and P in post-smolt Atlantic salmon [9]. An enhanced gut net-absorption of Ca and a higher Ca retention was also found in rats (*Rattus norvegicus*, Sprague Dawley outbred type) subjected to chronic respiratory acidosis caused by 14 days of treatment with high CO_2_ [44]. 

Studies which consider the synergistic effect of high CO_2_ and dietary P on the systemic response of Atlantic salmon and bone mineralization remain scarce [1,46]. This study analyses the effect of three levels of total dietary P: 0.5P (6.3 g/kg), 1P (9.0 g/kg), or 3P diet (26.8 g/kg) on the skeletal health of Atlantic salmon post-smolts reared in a land-based, flow-through system with seawater which was not injected with CO_2_ and contained a regular CO_2_ level (5 mg/L, 5CO_2_) or in seawater with injected CO_2_ thus raising the level to 20 mg/L (20CO_2_). The effect of high level water CO_2_, an environmental condition that is increasingly common on fish farms, was studied in the context of a possible partial compensation for reduced dietary P. An oversupplementation of Atlantic salmon with dietary P is discussed for its adverse effect on growth and feed intake. 

## 2. Materials and Methods

### 2.1. Animal Stock

The study was performed in agreement with Norwegian legislation and approved by the Norwegian Food Safety Authority (FOTS ID 25657). The trial was carried out at Skretting Aquaculture Innovation Lerang Research Station (Forsand, Norway) using the Atlantic salmon strain from Benchmark Genetics (Bergen, Norway). From the start of feeding, the animals were kept in 2 × 2 m square (2900 L) tanks. At an average weight of 7.9 g, the animals were moved to 5 m circular (32,000 L) tanks. The animals were kept in freshwater tanks with continuous light regime 24:0 (light: dark) at temperatures ranging from 5.55 °C in December 2019 to 14.35 °C in August 2020. To facilitate smoltification, the light regime was set to 12:12 (light:dark) to simulate ‘winter’, 9.5 weeks before seawater transfer followed by continuous light regime 24:0 (light:dark) to simulate ‘spring’, 3.5 weeks before seawater transfer [47,48]. Atlantic salmon were thereafter exposed to a continuous light regime. The animals were vaccinated in September 2020 (Alpha Ject micro 6; Pharmaq, Norway) and transferred to seawater in November at an average weight of 97 g. Prior to the experiment, the animals were kept in 8 m flow-through seawater tanks (70,000 L) at 12 °C and fed a standard commercial diet (Spirit Trout, Skretting) twice a day. 

### 2.2. Experimental Period

**Figure 1:**
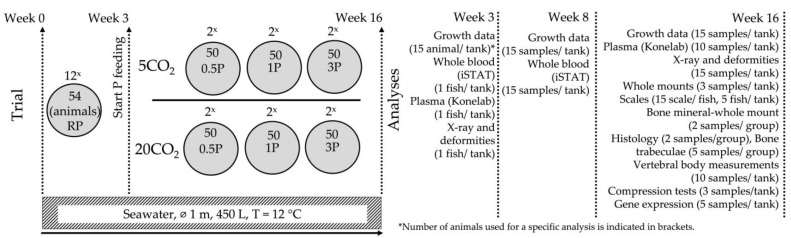
Experimental set-up and analysis. Animals were acclimatized for three weeks prior to phosphorus (P) feeding. Atlantic salmon were subsequently fed different levels of total phosphorus (P) 0.5P (6.3 g/kg), 1P (9.0 g/kg), and 3P (26.8 g/kg) and reared in conditions with no CO_2_ injected (5 mg/L) (5CO_2_) or with injected CO_2_ (20 mg/L) (20 CO_2_) for 13 weeks (weeks 3–16) in seawater. Each treatment was made in duplicates. Analyses, types, and number of samples taken at specified time-points are indicated.

Atlantic salmon were sedated (Aqui-S vet.; MSD, Bergen, Norway) and transferred to 450 L tanks with 1 m diameter (week 0) for an acclimatization period of three weeks (Figure 1). After three weeks of acclimatization, all fish were anaesthetized with MS-222 (Tricaine methane sulphonate; Pharmaq, Oslo, Norway) and graded to achieve an even weight within and between tanks at the start of the trial. The experimental period started at week 3 (Figure 1). The maximum animal density in seawater was 20.6 kg/m^3^. The animals were kept at 12 °C and a water current velocity of 0.8 L kg^−1^ min^−1^. For the P feeding experiment (weeks 3–16), semi-purified isocaloric and isonitrogenous diets were prepared to contain three different levels of total P: 0.5P (6.3 g/kg), 1P (9.0 g/kg), and 3P diet (26.8 g/kg) (Skretting Aquaculture Innovation Feed Technology Plant) (Table 1). The animals were fed three times a day. Dietary P levels were met by adjusting the supplementation of inorganic P in the form of mono-ammonium phosphate (MAP) (Table 1). Next to mono-sodium phosphate, MAP represents a source of inorganic phosphates with the highest mineral digestibility for salmonids leading to the lowest total P losses [49,50,51]. Commercial diets use predominantly MAP as a source of dietary P, thus it was used in this experiment. The control diet (1P) was formulated to meet the requirement for total P in the early seawater stage of Atlantic salmon (8.8–11.0 g/kg total P) [52]. While the 0.5P diet was formulated without supplementation of MAP, the 3P diet was formulated to contain an excessive level of dietary P comparable to high levels of total P used in Fjelldal et al. (21.5 g/kg) [53] and in Smedley et al. (19.7 g/kg) [54]. This is to understand the effect of high dietary P on bone formation and bone mineralization in Atlantic salmon. Targeted levels of total P were met whilst keeping the remaining feed raw materials constant by use of cellulose as a filler (Table 1). Cellulose is commonly used as a filler material and nutrient substitute in experimental diets with little or no nutritional value thus assuming that cellulose is inert in Atlantic salmon [55,56]. In this experiment up to 9.22% of cellulose (0.5P diet) was used to maintain a constant rate of raw ingredients while increasing the content of MAP. In a study by Kraugerud et al. [57], inclusion of 100 g/kg of cellulose in a diet for Atlantic salmon did not significantly affect macronutrient or P apparent digestibility compared to a fishmeal control diet. Similarly, Hansen and Storebakken [58] did not find any effects of increasing dietary cellulose level ranging from 0 to 150 g/kg on the digestion of main nutrients or fecal excretion of P in rainbow trout. Bromley and Adkins [59] showed that a diet containing up to 300 g/kg dietary cellulose does not have an adverse effect on the animal’s nutrient intake and growth rate. To monitor feed intake, excess feed was collected once a day by feed collectors with a mesh size of <2 mm. Carbon dioxide was added to the tanks by ceramic diffusors (Gas-X, Storvik Aqua AS, Sunndalsøra, Norway). The level of CO_2_ in 5CO_2_ tanks was measured at the beginning and end of the trial (3–5 mg/L) with a portable CO_2_ analyzer (OxyGuard, Farum, Denmark). The level of CO_2_ in 20CO_2_ tanks was measured once a day and averaged 22.61 ± 1.25 mg/L. The temperature and oxygen levels were monitored daily at a depth of 1 m and averaged 12 ± 0.15 °C and 102.35 ± 4.77%. The level of salinity was kept at 34 ppt. The pH measured at the end of the trial (pH meter WTW, pH3110; Sentix 41) was 7.94 in a tank without injected CO_2_ and 7.21 in a tank with injected CO_2_.

### 2.3. Sampling and Growth Measurements

The animals’ fork length, weight, and condition factor were measured (15 animals/tank, 30 animals/group) (at week 3, 8, and 16 (Figure 1)). Fulton’s condition factor (CF) was calculated according to the following equation:

CF = (W × L^−3^) × 100, (1)

where W is weight (g) and L is fork length (cm) [61]. The specific growth rate (SGR) was calculated as follows:

SGR = (% body mass day^−1^) = [(W_week16_/ W_week3_)^1/Days^ − 1] × 100, (2)

where W_week3_ and W_week16_ represent the average animal weight for a given period and days represent the number of feeding days for that period [4]. The feed conversion ratio (FCR) was calculated on a dry matter basis (FCR = total feed consumed/weight gain) [60]. Feed intake (g of feed day^−1^) was recorded daily based on the quantity of recovered feed subtracted from the total daily ration. 

### 2.4. Blood Analysis

Blood was collected at weeks 3, 8, and 16 after a 24 h starvation period by puncture of the ventral caudal peduncle in 4 mL lithium heparinized Vacuette tubes (Greiner Bio-One GmbH, Kremsmünster, Austria) and placed on ice. Whole blood samples were analyzed by an iSTAT hand-held Portable Clinical Analyzer with an i-STAT G3+ cartridge (Abaxis Europe GmbH, Griesheim, Germany) on a VetScan iSTAT1 handheld blood analyzer (Abaxis Europe GmbH, Griesheim, Germany) at weeks 3 and 8. The remaining whole blood samples were centrifuged at 4000× *g* (4 °C) for 7 min with a VWR Mega star 600R centrifuge (Darmstadt, Germany), the plasma supernatant was carefully collected, and stored at −80 °C. Plasma was chemically analyzed on a Konelab 30i chemistry analyzer (Thermo Fisher Scientific, Basel, Switzerland) at weeks 3 and 16 according to the manufacturer’s instructions.

#### 2.4.1. Whole Blood Analysis

For the iSTAT analysis, blood was collected from 24 animals at week 3, and from 5 animals/ tank (10 animals/ group) at week 8 (Figure 1). A volume of 120 µL of whole blood was analyzed for pH, PCO_2_ (partial pressure of CO_2_, i.e., the amount of CO_2_ dissolved in blood), PO_2_ (partial pressure of O_2_), bicarbonate ions, TCO_2_ (total CO_2_), and sO_2_ (O_2_ saturation, i.e., the fraction of hemoglobin able to bind to O_2_). 

#### 2.4.2. Blood Plasma Analysis

Samples for blood plasma were collected from 10 animals/tank (20 animals/group) (Figure 1). The concentration of total and non-bound Ca^2+^, Cl^−^, inorganic P (P*i*), pH, Zn^2+^, ALP, and bicarbonate ions were measured by the Skretting Aquaculture Innovation Laboratory (Stavanger, Norway). The non-bound Ca^2+^, Cl^−^, and P*i* were determined as described in [39]. The total Ca^2+^ and pH was measured by ion selective electrode (art. #981772, #981597, respectively). Photometry at 560 nm was applied for quantitative determination of Zn^2+^ and the use of 5 Br-PAPS. Photometry at 405 nm was used for quantitative determination of bicarbonate ions and ALP levels (art. #981889, #981833, respectively). Alkaline phosphatase activity (ALP) was determined in three animals/tank (six animals/group) using p-nitrophenyl-phosphate (4-NPP) as a substrate in a 2-amino-2-methyl-1-propanol (AMP) phosphate-accepting buffer. The ALP activity was expressed as enzyme units (U/L). The activity of bone/liver/kidney ALP (b/l/k ALP) was determined with the application of 10 mmol levamisole (VWR Chemicals) to inhibit the non-intestinal form of the enzyme [62,63]. The measured activity of intestinal ALP was then deducted from total plasma ALP to determine b/l/k ALP. 

### 2.5. Radiography

Animals were filleted on both sides and placed on a 10 MP digital X-ray tablet and radiographed by a portable X-ray unit Gierth TR 90/30 peak (Riesa, Germany). Radiographs were taken at 80 cm distance between the X-ray source and the tablet at 40 kV, 0.2 mA, and 0.2 s exposure time. Radiographs were analyzed as Dicom files using RadiAnt DICOM viewer (Medixant, Poznań, Poland). A sample of 24 animals was X-rayed at week 3 to check for deformities at the start of the trial. At week 16, 15 animals/tank (30 animals/group) were X-rayed (Figure 1).

### 2.6. Vertebral Centra Deformity Diagnosis

**Figure 2:**
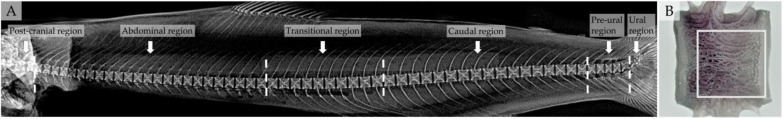
Vertebral column regions and the location of vertebral centra used for gene expression analysis. (**A**) Regions of the vertebral column in Atlantic salmon demarcated by white dashed lines; (**B**) Rectangle specifying the location of vertebral centrum (from the transitional vertebral region, below the dorsal fin) which was sampled for gene expression analysis.

Deformities were analyzed based on digital X-ray images. Vertebral column regions were identified following (Figure 2A) [64]. Types of vertebral deformities were recorded based on the types described by [65]. The fusion of one or two vertebrae to the base of the skull (occipital region) or one or two vertebrae to the urostyle was not included in the deformity count. These fusions are common, non-pathological phenomena in wild and farmed teleosts [66,67,68,69,70]. 

### 2.7. Vertebral Body and Scale Morphology

For the histological and whole mount staining analyses, vertebral columns were fixed in 10% buffered formalin for at least 72 h, rinsed with tap water three times, and left in tap water overnight while the water exchange was achieved using a dripping tap. Samples were then transferred stepwise to 70% ethanol until further analysis. Scales (~15 scales/animal) were collected from the area between the adipose fin and the lateral line [71] and stored in acetone (VWR, Darmstadt, Germany) at −20 °C.

#### 2.7.1. Whole-Mount Alizarin Red S Staining 

Whole-mount Alizarin Red S staining for mineralized bone was performed on the caudal vertebral region (Figure 2A) in three samples/tank (six samples/group) (Figure 1) according to [38]. Vertebral bodies were analyzed for structure and, based on the extent of mineralization, vertebral bodies were characterized as low-mineralized or mineralized.

**Figure 3:**
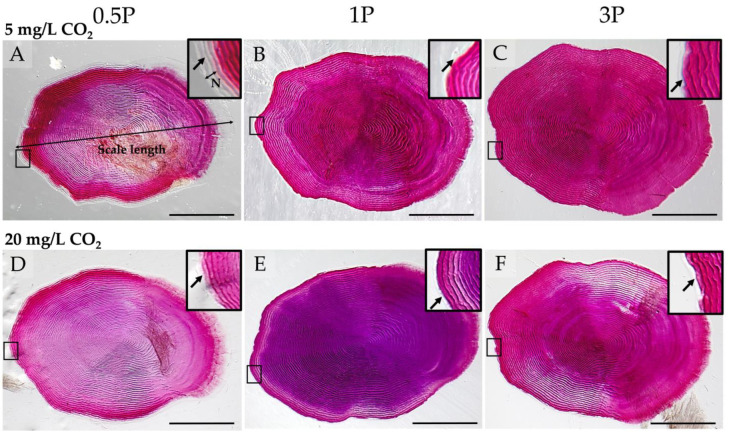
Scale morphometry. Representative samples of scales stained with Alizarin Red S from animals fed different levels of total phosphorus (P) 0.5P (6.3 g/kg) (**A**,**D**), 1P (9.0 g/kg) (**B**,**E**), and 3P (26.8 g/kg) (**B**,**F**), and reared in conditions with no CO_2_ injected (5 mg/L) (**A**–**C**) or with injected CO_2_ (20 mg/L) (**D**–**F**). Red staining represents the mineralized part of the scale (**A**–**F**) while the non-mineralized area of the scale (N) (black arrows in rectangles in **A**–**F**) remains unstained. Total length (L) of the scale is indicated (**A**).

Scales from five fish/tank (10 fish/group) (Figure 1) were bleached in 3% H_2_O_2_ and 1% KOH for 4 h and stained with 0.5% Alizarin Red S solution for 15 min, rinsed in dH_2_O, and gradually transferred into 100% glycerol through a 1% KOH-glycerol series, as described in [41]. Scale length and length of the non-mineralized part of the scale were measured (Figure 3A). Observations on the vertebral column and scales were made using oblique illumination on a Zeiss Axio Zoom V16 Fluorescence Stereo Zoom Microscope equipped with a 5MP CCD camera for imaging.

#### 2.7.2. Histology

Vertebral bodies no. 32–35 and 36–39 were dissected for serial sectioning of previously non-demineralized or demineralized samples (two samples/group) (Figure 1). For embedding in glycol metacrylate (GMA), samples were treated as described in [38]. Sections were stained with toluidine blue, a metachromatic stain which visualizes skeletal and cartilaginous tissues, cellular structures, and collagen fibers. von Kossa/Van Gieson was used to visualize phosphate in the mineralized bone and type I collagen bone matrix [72]. Sections were cover slipped with DPX (Sigma-Aldrich, Overijse, Belgium) and observed using a Zeiss Axio Imager-Z compound microscope (Carl Zeiss Microscopy GmbH, Jena, Germany) equipped with an Axiocam 503 color camera. 

### 2.8. Mineral Analysis of Vertebral Column

Vertebral columns were stained with Alizarin Red S to determine the extent of mineralization prior to bone mineral analysis. Individual Alizarin Red S stained vertebral columns of animals fed the 0.5P and 1P diet were analyzed for bone mineral content to determine if the content differs between animals reared in 5 mg/L CO_2_ and 20 mg/L CO_2_. One sample/tank (two samples/group) were analyzed (Figure 1). Samples were gradually rehydrated stepwise to dH_2_O through glycerol series (3:1, 1:1, 1:3), 1 h per step, analysis samples were then rinsed with distilled water (dH_2_O). Lipids were removed in a 1:1 (*v*/*v*) solution of acetone and methanol (2 × 8 h). Samples were dried for 24 h at 105 °C [40]. Samples were processed and analyzed according to [38]. Samples were analyzed by Masterlab Analytical Services (Nutreco, Boxmeer, The Netherlands). Only 0.5P/20 animals characterized as mineralized were analyzed in 0.5P/20 mg/L group. Animals fed 3P were not analyzed as the analysis of X-ray images, whole mount Alizarin Red S stained specimens, and non-demineralized histological section in the current study and in other studies [39,40,41] showed no differences between the mineral content and extent of bone mineralization between Atlantic salmon fed regular and high P in freshwater and seawater.

### 2.9. Vertebral Body Measurements

**Figure 4:**
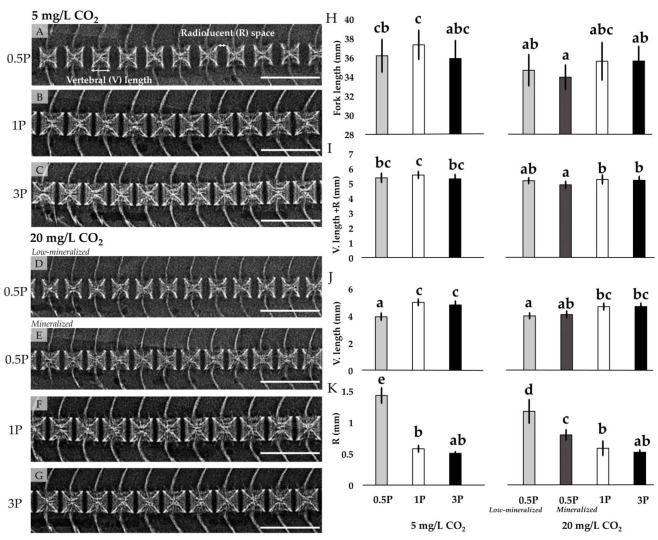
Radiography. X-ray images, scale bar = 1.5 cm, show representatives of vertebral centra of animals fed different levels of total phosphorus (P) 0.5P (6.3 g/kg), 1P (9.0 g/kg), and 3P (26.8 g/kg) and reared in conditions with no CO_2_ injected (5 mg/L) or with injected CO_2_ (20 mg/L) (**A**–**G**); Vertebrae were analyzed for the extent of vertebral (V) length and radiolucent (R) space (**A**); Vertebral bodies in 15 out of 30 animals fed 0.5P diet and reared in CO_2_ 20 mg/L were mineralized. Significant differences in measurements are denoted by a different letter. Fork length and V. length + R in mineralized 0.5P/20CO_2_ animals were significantly reduced compared with 0.5P/5CO_2_ animals (**H**,**I**); Vertebral length was significantly reduced in 0.5P/5CO_2_ and 0.5P/20CO_2_ low-mineralized animals (**J**); Radiolucent space was enlarged in 0.5P/5CO_2_ animals. The low-mineralized phenotype was partially recovered at high CO_2_ rearing conditions (**K**).

Measurements were taken from digital X-ray images with a RadiAnt Dicom Viewer at week 16 (Figure 1). Vertebral centrum anterior–posterior length (radiopaque area), and the radiolucent distance (Figure 4A) were measured in 10 animals/tank (20 animals/group) (Figure 1) on vertebral bodies no. 32–41 following [39].

### 2.10. Vertebral Centrum Compression Tests

The compression tests were carried out at the Institute of Marine Research, Matre Research Station, Matredal, Norway on three vertebrae/ sample in three animals/tank (six samples/group) (Figure 1). Vertebra no. 33–35 from the transitional region (Figure 2A), a region with higher stiffness and yield load [48,73], were dissected. Neural and hemal arches and the remains of notochord tissue were removed and vertebrae were stored in saline solution (6 ppt), at 4 °C. Vertebral centra were measured for anterior–posterior length, dorsal–ventral length, and lateral length diameters to the nearest 0.01 mm using a slide caliper. Load deformity testing was measured with a texture analyzer (Model: TA-HD plus Texture Analyzer; Stable Micro Systems Ltd., Surrey, UK) by compressing a single vertebral centrum along its anterior–posterior axis with a steadily advancing piston (0.01 mm/s). The test was terminated at 35% compression. The load deformity data were used to calculate the mechanical properties adjusted for vertebra size/dimensions [74]. The Texture Exponent Software Version 6 (Stable Micro Systems Ltd., Surrey, UK) was used for calculations of values. Size/dimension-adjusted mechanical properties (modulus of elasticity, yield point, failure point, and toughness) were calculated using a modified version of [74], where the yield point was determined by a 0.2% offset line [75], and the failure point was determined by a 5% offset line according to [76]. 

### 2.11. Gene Expression Analysis

Bone tissue of the vertebral centra (the rectangle in Figure 2B) was collected from the anterior transitional region (below the dorsal fin) (Figure 2A) (one vertebra/animal, five vertebrae/tank, and ten vertebrae/animal group) (Figure 1). Samples were cleaned of muscle, kidney tissue, blood vessels, spinal cord, notochord tissue, and stored in RNA-later, at −80 °C. For RNA extraction, 26–32 mg of vertebral tissue from a single vertebra sample was homogenized in 350 µL of RTL lysis buffer (RNeasy Mini Kit, Qiagen, Hilden, Germany) with 5 mm stainless steel beads in a TissueLyser (Qiagen, Hilden, Germany) (2 cycles of 2 min, 25 Hz). A total of 20 µL of RNA was isolated with the RNAeasy Fibrous Tissue Mini Kit (Qiagen) on a QIAcube workstation. DNase treatment was applied (RNAase free DNase set (250), Qiagen, Hilden, Germany). RNA quality and concentration were determined by a NanoDrop spectrophotometer (Thermo Fisher Scientific Inc, Verona, WI, USA). The RNA was reverse transcribed in a thermocycler (T100 Thermal Cycler; BIO-RAD) with the QuantiTect Reverse Transcription kit (Qiagen, Hilden, Germany) Real-Time PCR was performed with SYBRgreen (PowerUp SYBR Green Master Mix; AppliedBiosystems by Thermo Fisher Scientific, Vilnius, Lithuania) on a Quant Studio 5 thermocycler (Thermo Fisher Scientific) which was programmed to: 50 °C for 2 min, 95 °C for 2 min (with a temperature ramp of 2.74 °C/s), 40 cycles consisting of 95 °C for 1s, and 60 °C for 30 s; this was followed by 95 °C for 1 s, 60 °C for 20s, and 95 °C for 1 s with a temperature ramp of 0.15 °C/s for melt-curve analysis. Samples were run in duplicates. Systematic negative controls containing no cDNA (NTC, no template control) were included. No amplification occurred in NTC. The analysis of the melt-curve showed a single peak for all reactions and an additional peak for several samples analyzed for the expression of *enpp1*. These PCR products were subsequently analyzed by gel electrophoresis. No additional products and no primer-dimers were detected in these products. The primers used to amplify the transcripts of *fgf23*, *entpd5*, and *enpp1* were designed by PRIMER3 (NCBI) (Table 2). Primer pairs used to amplify transcripts of *bgp*, *col1a1a*, and *opn* were obtained from [77] (Table 2). Target specificity was checked in silico using Blast (NCBI). Only primer pairs with no unintended targets were selected. The relative gene expression was calculated by the ΔΔCt method [78] using *β-actin* and *ef1a* as the reference genes [79,80]. Reference genes were selected using RefFinder [81]. To determine the efficiency of each primer pair standard curves were performed in triplicates using a series of 5-fold cDNA dilutions (1:5, 1:25, 1:125, 1:625, 1:3125).

The target genes analyzed in the present study were selected based on their function of the respective proteins in bone mineralization, bone formation, and P metabolism. Type I collagen is the most prominent collagen in teleost skeleton [82]. Based on a study in zebrafish collagen in teleosts consists of three pro-alpha chains α1(I), α3(I), and α2(I) coded by *col1a1a*, *col1a1b*, and *col1a2*, respectively [82]. *col1a1a* thus represents a specific marker for collagen type I. Osteocalcin/ Bone gla protein (BGP) and osteopontin (OPN) represent abundant non-collagenous proteins in bone. The exact function of BGP is still being discussed. *Bgp* is synthetized by osteoblasts and can thus be used as a reliable marker for bone matrix formation [83]. OPN is supposed to inhibit bone mineralization and has also been suggested to be required for osteoclastic bone resorption [84,85]. Ectonucleotide pyrophosphatase phosphodiesterase 1 (*enpp1*) and ectonucleoside triphosphate/diphosphohydrolase 5 (*entpd5*) are reciprocal regulators of bone mineralization by determining the level of pyrophosphate, the most potent bone mineralization inhibitor [86]. ENPP1 is a positive regulator of pyrophosphate synthesis [87]. ENTPD5 hydrolysis extracellular nucleotide diphosphates into nucleotide monophosphates and inorganic phosphate and hence represents a positive regulator of bone mineralization [86,88]. Fibroblast growth factor 23 (*fgf23*) is a hormone produced by osteoblasts and osteocytes. Increased levels of FGF23 suppress the renal phosphate reabsorption [89].

### 2.12. Statistical Analyses

All data are given as mean ± standard deviation. Data for gene expression are given as mean ± standard error. The data were arranged by a two factorial arrangement, with two levels of CO_2_ (5CO_2_ and 20CO_2_) and three levels of dietary P (0.5P, 1P, and 3P) and analyzed with a general linear model (GLM). Variances among groups were analyzed using one-way ANOVA followed by Tukey’s post hoc test (Bonferroni corrected) or Kruskal–Wallis test with pairwise post hoc comparisons (Bonferroni corrected). The Shapiro–Wilk test was used to test the normal distribution of the variances. Values for week 3 were not included in the statistical analysis. The SPSS software was used for the statistical analysis (Version 27, IBM Corp., Armonk, NY, USA). Individual animals were considered as statistical replicates. For SGR, FCR, feed intake, and total feed eaten tanks were considered as statistical replicates and variances among groups were therefore not analyzed. The level of statistical significance was set at *p* ≤ 0.05. 

## 3. Results

### 3.1. Diets

The current semi-purified diets were formulated to differ in the content of P. The proximate analysis showed that the targeted total and estimated available P in the diets were reached (Table 1). Contents of moisture, crude protein, and crude fat were comparable. Ash and total iron (Fe) content in the diets increased gradually with the increased content of MAP (Table 1). 

### 3.2. Growth Data

The data on fork length, weight, and condition factor at week 3 are listed in Table 3. Values for fork length, weight, and condition factor at weeks 8 and 16 and data on SGR, FCR, total amount of eaten feed, and feed intake at week 16 with significant explanatory variables for these parameters can be found in Table 3. Total feed intake, total feed eaten, and all growth-related parameters except FCR were reduced in the 3P animal group and in all dietary P animals reared in 20 mg/L CO_2_ (Table 3). The FCR was elevated in animals from all dietary P groups reared in 20 mg/L CO_2_ (Table 3). High CO_2_ and interaction between high CO_2_ and dietary P (0.5P and 3P) resulted in a reduced fork length and weight in week 8, and weight and condition factor in week 16 (Table 3).

### 3.3. Mineralization

#### 3.3.1. Radiology

X-ray images only show the mineralized parts of the vertebral bodies. Vertebral body endplates can be recognized as typical X-shaped structures with radiolucent spaces between the mineralized vertebral centra (Figure 2A). The radiolucent spaces (R) between the vertebral centra were significantly extended in 0.5P/5CO_2_ (Table 4). Vertebral length + R and fork length of animals used for vertebral body measurements was significantly reduced in 0.5P/20CO_2_. Vertebral length was significantly reduced in 0.5P, irrespective of the CO_2_ level (Table 4). A low-mineralized phenotype characterized by reduced vertebral length and vertebral height (radiodense area) and extended radiolucent space between the mineralized parts of the vertebral bodies was found in all 0.5P/5CO_2_ animals. At week 16, 15 out of 30 animals fed the 0.5P diet and reared in 20 mg/L CO_2_ were mineralized, while the remaining 15 animals were low-mineralized (Figure 4). Further measurement of the low-mineralized and mineralized 0.5P/20CO_2_ animals revealed that the extent of R was significantly reduced in the mineralized 0.5P/20CO_2_ group compared with the 0.5P/5CO_2_ and low-mineralized 0.5P/20CO_2_ group (Figure 4). Measurements of the mineralized part of the vertebral centra showed no differences between the 1P and 3P diet groups irrespective of the CO_2_ level (Figure 4). 

**Table 4:** Vertebral centra measurements. The analysis was performed on X-ray images of the vertebral column in animals fed diets containing different levels of total P 0.5P (6.3 g/kg), 1P (9.0 g/kg), and 3P (26.8 g/kg) which were reared in conditions with no CO_2_ injected (5 mg/L) or with injected CO_2_ (20 mg/L). Fork length of animals used for this analysis and vertebral (V) length + radiolucent space (R) were reduced in 0.5P/20CO_2_ animals. The extent of vertebral length was reduced in 0.5P animals irrespective of the CO_2_ treatment. Radiolucent space was significantly increased in 0.5P/5CO_2_ animals. The statistically significant differences among groups (within a row) are indicated by different lowercase superscript letters. If none of the letters are the same, differences are significant. The significant values (*p*) for CO_2_, diet (D), and their interaction are indicated.

CO_2_	5 mg/L CO_2_			20 mg/L CO_2_			*p*-Value		
**Diet/** **Measurements**	**0.5P**	**1P**	**3P**	**0.5P**	**1P**	**3P**	**D**	**CO**_**2**_	**D × CO**_**2**_
**Fork length (mm)**	36.18 ± 1.71 ^bc^	37.32 ± 1.52 ^c^	35.89 ± 1.87 ^bc^	34.30 ± 1.48 ^a^	35.61 ± 1.96 ^ab^	34.96 ± 1.53 ^ab^	**0.002**	**<0.001**	0.388
**V. length + R (mm)**	5.38 ± 0.25 ^bc^	5.56 ± 0.24 ^c^	5.31 ± 0.30 ^bc^	5.04 ± 0.26 ^a^	5.26 ± 0.34 ^ab^	5.21 ± 0.26 ^ab^	**0.002**	**<0.001**	0.105
**V. length (mm)**	3.95 ± 0.34 ^a^	4.99 ± 0.24 ^c^	4.80 ± 0.29 ^bc^	4.11 ± 0.24 ^a^	4.68 ± 0.32 ^b^	4.69 ± 0.26 ^b^	**<0.001**	0.080	0.318
**R (mm)**	1.43 ± 0.23 ^c^	0.58 ± 0.05 ^b^	0.51 ± 0.03 ^b^	0.93 ± 0.23 ^ab^	0.59 ± 0.04 ^b^	0.52 ± 0.03 ^b^	**<0.001**	**<0.001**	**<0.001**

#### 3.3.2. Mineralization Pattern

**Figure 5:**
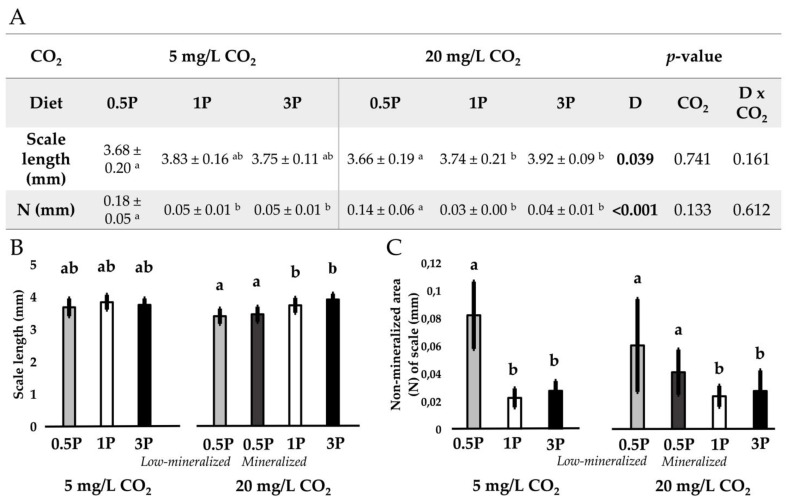
Scale measurements. Values for scale length and non-mineralized area (N) of scale in animals fed different levels of total phosphorus (P) 0.5P (6.3 g/kg), 1P (9.0 g/kg), and 3P (26.8 g/kg) and reared in conditions with no CO_2_ injected (5 mg/L) or with injected CO_2_ (20 mg/L) (**A**). The statistically significant differences among groups (within a row) are indicated by different lowercase superscript letters. If none of the letters are the same, differences are significant. The significant values (*p*) for CO_2_, diet (D), and their interaction are indicated. (**A**,**B**) show average values for scale length and N which include measurement of 0.5P animals reared in 20 mg/L CO_2_ which showed either low-mineralized or mineralized bone phenotype. While the scale length of 0.5P/20CO_2_ mineralized animals is comparable to 0.5P/20CO_2_ low-mineralized (**B**), 0.5P/20CO_2_ mineralized animals show a trend to reduce the extension of N in the anterior part of scales (**C**). Significant differences in measurements are denoted by a different letter.

**Figure 6:**
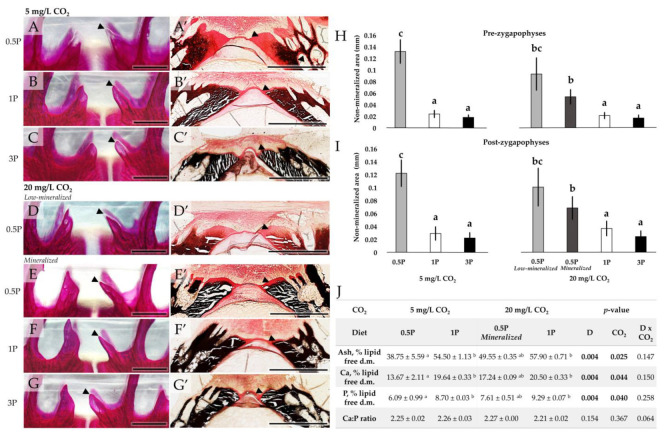
Mineralization pattern and Alizarin Red S mineral analysis. Whole mount Alizarin Red S stained (**A**–**G**) and non-demineralized sections (**A’**–**G’**) (von Kossa/Van Gieson), scale bar = 1 mm of representative vertebral bodies from animals fed different levels of total phosphorus (P) 0.5P (6.3 g/kg), 1P (9.0 g/kg), and 3P (26.8 g/kg) and reared in conditions with no CO_2_ injected (5 mg/L) or with injected CO_2_ (20 mg/L). Black arrowheads in (**A**–**G’**) point to the areas of non-mineralized bone at the pre-zygapophyses (**A**–**G**) and at the vertebral body endplates (**A’**–**G’**). (**A**–**A’**) Representative samples from animals fed 0.5P and reared in 0.5 mg/L CO_2_ showing increased areas of non-mineralized bone at the zygapophyses and vertebral body endplates. (**E**,**E’**) Representative samples from animals fed 0.5P and reared in 20 mg/L CO_2_ showing reduced extent of the non-mineralized bone at the pre-zygapophyses and vertebral body endplates. Graphs show the extent of the non-mineralized area of bone at the pre-zygapophyses (**H**) and post-zygapophyses (**I**). Different letters indicate significant differences among the animal groups. Table shows bone mineral content analysis. The statistically significant differences among groups (within a row) are indicated by different lowercase superscript letters. If none of the letters are the same, differences are significant. The significant values (*p*) for CO_2_, diet (D), and their interaction are indicated. There was a trend of an increased ash, Ca, and P (**J**) in the vertebral column of animals reared in high CO_2_ and fed increased dietary P content.

Alizarin Red S stained specimens and non-decalcified parasagittal histological sections from 0.5P/5CO_2_ and from low-mineralized 0.5P/20CO_2_ animals demonstrated extended areas of non-mineralized bone matrix at the edges of the anterior part of scales (Figure 3A and Figure 5), bone trabeculae, vertebral body endplates, and zygapophyses (Figure 6A–A’,D–D’,H,I, Table 5). The areas of low-mineralized bone at zygapophyses and vertebral body endplates were smaller in mineralized 0.5P/20CO_2_ (Figure 6E–E’,H,I) but remained significantly larger compared with the animals of the 1P/5CO_2_, 1P/20CO_2_, 3P/5CO_2_, and 3P/20CO_2_ groups (Figure 6B–C’,F–G’,H,I). Samples from these animals showed a regular thin layer of non-mineralized bone (osteoid) at the distal edges of scales (Figure 3B,C,E,F and Figure 5A,C), vertebral body endplates, and zygapophyses (Figure 6B–C’,F–G’).

#### 3.3.3. Mineral Analysis

The values for ash, Ca, and P analysis on Alizarin Red S stained samples revealed a gradual increase in bone mineral content from the lowest values in 0.5P/5CO_2_ animals, intermediate values in 0.5P/20CO_2_, and the highest values in 1P/5CO_2_ and 1P/20CO_2_ samples (Figure 6J). The content of ash in the vertebral column was significantly affected by different dietary P content and by high water CO_2_. The Ca:P ratio remains comparable among all animal groups (Figure 6J).

### 3.4. Vertebral Centra Deformities

Deformities were not observed in the samples collected at week 3. There were no differences in the vertebral centra deformity prevalence among diet groups, neither was there an effect of elevated CO_2_ on the increased rate of deformities at week 16. Mild deformities were found in five 0.5P/5CO_2_, two 1P/5CO_2_, four 3P/5CO_2_, four 0.5P/20CO_2_, four 1P/20CO_2_, and two animals in the 3P/20CO_2_ animals. Regions of the vertebral column affected with deformities were the abdominal and transitional regions (Figure 2A for regions) (17 deformed animals), and the caudal region (8 deformed animals). The average number of deformed vertebrae per deformed animal was 3.53 ± 3.1. Deformity types were compression- and fusion-related: compression with fusion, one-sided compression, fusion centra, complete fusions, and homogenous compression. Hyper-dense vertebrae, characterized by an increased radiodensity on X-ray images, were found in five animals and one animal had a vertical shift with deformed intervertebral joints. 

### 3.5. Vertebral Body Morphology and Structure

**Figure 7:**
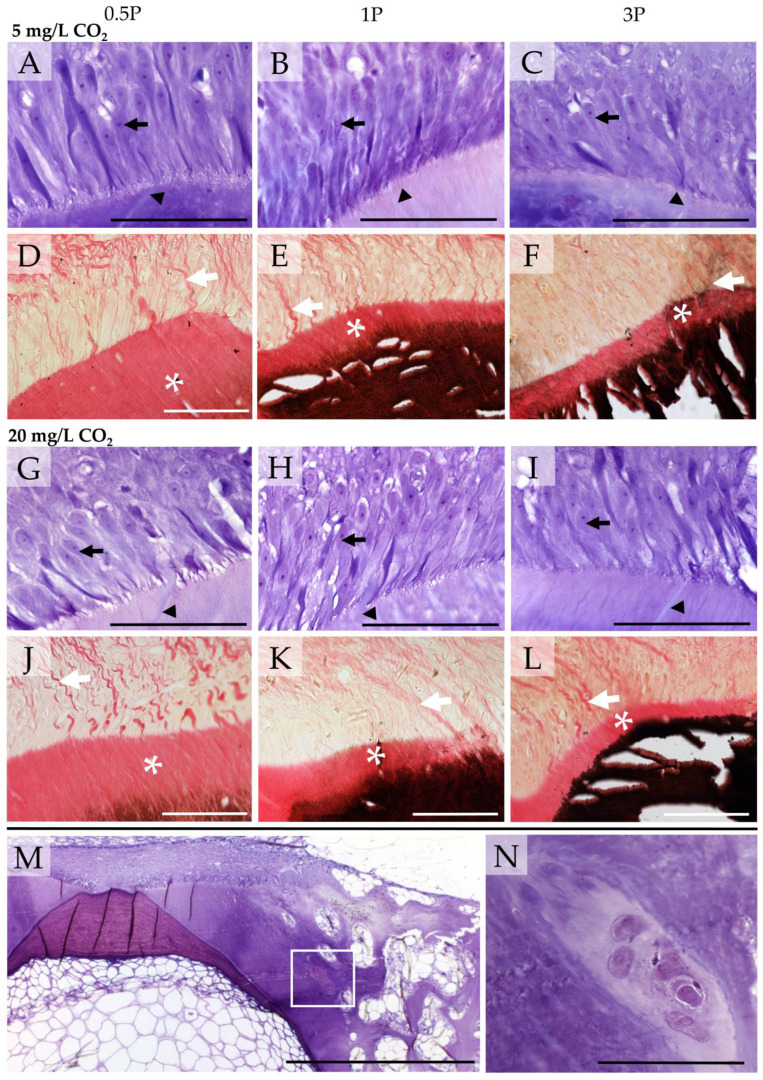
Morphology of vertebral bodies. (**A**–**C**,**G**–**I**) Demineralized parasagittal sections show a detailed depiction of active spindle-shaped osteoblasts (black arrows) and Sharpey’s fibers (black arrowheads) in representative samples of Atlantic salmon at week 16 fed different levels of a total phosphorus (P) 0.5P (6.3 g/kg), 1P (9.0), and 3P (26.8 g/kg) and reared in conditions with no CO_2_ injected (5 mg/L) or with injected CO_2_ (20 mg/L); (**D**–**F**,**J**–**L**) Non-demineralized parasagittal sections depict type I collagen fibers (white arrows) and the extension of non-mineralized bone at the vertebral body endplates (white asterisks); (**M**) Demineralized parasagittal sections show ectopic cartilage between the distal part of the vertebral body endplates and the bone trabeculae (white rectangle); (**N**) A magnified view of such ectopic cartilage; (**A**–**C**,**G**–**I**,**M**,**N**) toluidine blue, (**D**–**F**,**J**–**L**) von Kossa/Van Gieson, (**A**–**L**,**N**) = 100 μm, M = 1000 μm).

Based on whole mount Alizarin Red S stained specimens and demineralized and non-demineralized serial histological sections, animals showed well-developed vertebral bodies in all diet groups (Figure 6A’–G’ and Figure 7). Osteoblasts were in active spindle-shaped form typical for salmon (black arrows in Figure 7A–C,G–I), collagen type I fiber bundles (white arrows in Figure 7D–F,J–L) and their extension in bone (Sharpey’s fibers) were present (arrowheads in Figure 7A–C,G–I). The vertebral centra of animals fed the 0.5P diet developed a characteristic ectopic cartilage at the interface between the non-mineralized vertebral body endplates and the trabecular bone region [40], which were present at the lateral side of the vertebral centra and absent from the midline of vertebral centra (white rectangle in Figure 7M,N). 

### 3.6. Blood Analysis

Whole blood analysis (iSTAT) from weeks 3 and 8 can be found in Table 6. The blood pH, PCO_2_, bicarbonate ions, and TCO_2_ were significantly increased in animals reared in 20 mg/L CO_2_ compared with animals reared in 5 mg/L CO_2_ (Table 6). The value for PCO_2_ was significantly increased by the 3P diet in animals reared in high CO_2_ compared with 0.5P/20CO_2_ animals (Table 6).

Blood plasma analysis (Konelab) for ALP, pH, bicarbonate ions, total and non-bound Ca^2+^, P*i*, and Zn^2+^ from weeks 3 and 16 can be found in Table 7. At week 16, plasma ALP increased with increasing dietary P and high 20 mg/L CO_2_ (Table 7). Plasma pH, bicarbonate ions, and total and non-bound Ca^2+^ were increased in 20 mg/L CO_2_ groups. Plasma Cl^−^ was significantly reduced by 20 mg/L CO_2_ (Table 7). Plasma P*i* showed a two-fold reduction in 0.5P/5CO_2_ and 0.5P/20CO_2_ relative to the other groups (Table 7). Plasma Zn^2+^ was significantly reduced by the 3P diet and 20 mg/L CO_2_ (Table 7).

### 3.7. Mechanical Properties of Vertebral Centra

The mechanical property analysis of the vertebral centra with associated significant differences between the treatments can be found in Table 8. Values for modulus of elasticity and failure point were significantly reduced by the 0.5P diet. Values for yield point and toughness in animals fed the 0.5P/5CO_2_ diet were significantly reduced compared with 3P/5CO_2_ and 3P/20CO_2_ (Table 8).

### 3.8. Gene Expression

**Figure 8:**
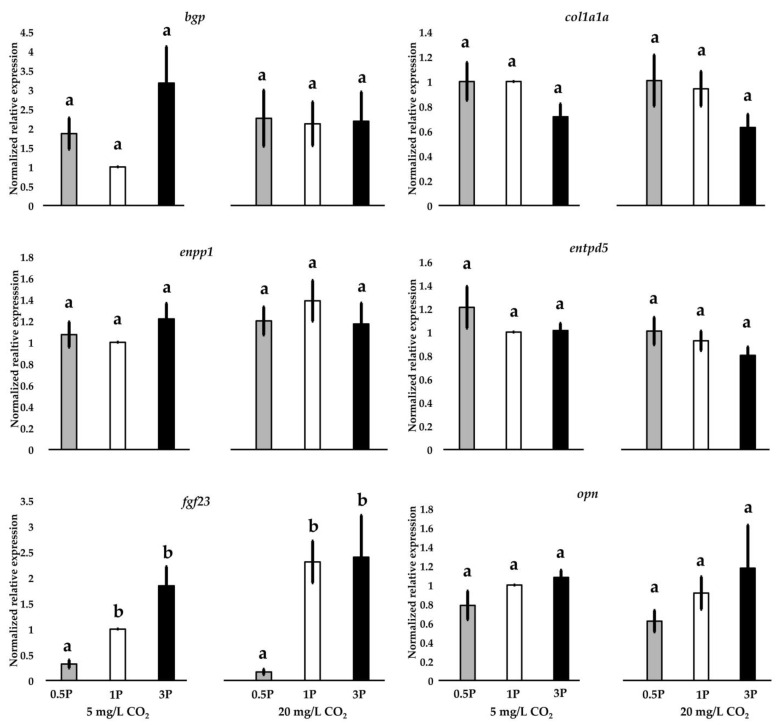
Gene expression. Quantitative PCR of gene expression in the vertebral centra of animals fed different levels of total phosphorus (P) 0.5P (6.3 g/kg), 1P (9.0 g/kg), and 3P (26.8 g/kg) and reared in conditions with no CO_2_ injected (5 mg/L) or with injected CO_2_ (20 mg/L). Graphs show expression of *bgp* (bone gla protein), *col1a1a* (collagen type 1 alpha 1a), *enpp1* (endonucleotide pyrophosphatase/phosphodiesterase 1), *entpd5* (ectonucloside triphosphate disphosphohydrolase 5), *fgf23* (fibroblast growth factor 23), and opn (osteopontin). *fgf23* expression levels were significantly downregulated in 0.5P/5CO_2_ animals compared with the other groups. Results are expressed as mean +/− standard error. Different letters denote significant differences among the groups.

Gene expression analysis showed a significant downregulation of *fgf23* expression in the 0.5P animals (Figure 8). A trend of downregulation of *fgf23* gene expression was observed in the 0.5P/20CO_2_ compared with 0.5P/5CO_2_ animals (Figure 8). Expression of the remaining genes associated with bone formation and bone mineralization (*bgp*, *col1a1a*, *enpp1*, *entpd5*, and *opn*) remained unaffected by the different levels of dietary P or level of CO_2_ (Figure 8).

## 4. Discussion

This study on Atlantic salmon analyzed the combined effects of dietary P and levels of dissolved CO_2_ in water on the mineralization of the vertebral column. The main findings that will be discussed below are: (i) an elevated level of CO_2_ in the water partly compensates the effect of a P deficient diet (0.5P), (ii) high dietary P and increased CO_2_ level, as single factors and in combination, reduce growth (SGR), (iii) independent from the water CO_2_ content, an increased level of dietary P does not improve bone mineralization. In addition, no effects of different dietary P levels on the occurrence of skeletal deformities are observed. 

### 4.1. Elevated CO_2_ Partly Compensates for Low Dietary P

The increase in bone mineralization is usually considered to be advantageous for Atlantic salmon and thus the dietary P content in commercial feeds is often elevated [53,90,91]. Increased levels of dietary P above the recommended 10.0 g/kg total P (1% total P) have been suggested as enhancing bone mineralization and preventing development of deformities in farmed Atlantic salmon [36,90]. Unexpectedly, in this study the Atlantic salmon fed a 0.5P diet showed comparable bone mineralization with animals fed a 1P and 3P diet when reared in water with 20 mg/L CO_2_ levels. In 15 out of 30 animals fed a 0.5P diet the typical low-mineralized phenotype (osteomalacia), characterized in Atlantic salmon and zebrafish by the development of extensive areas of non-mineralized bone [39,40,41,42,92], was completely absent. X-ray images of 0.5P/20CO_2_ showed reduced radiolucent areas between the radiodense parts of the vertebral centra. Analysis of whole mount Alizarin Red S specimens of vertebral bodies, scales, and serial histological sections revealed a thin layer of non-mineralized bone in the 0.5P/20CO_2_ group somewhat comparable to the thickness of the osteoid in the fully-mineralized 1P and 3P animals. The remaining 15 animals in 0.5P/20CO_2_ showed an intermediate level of the osteoid thickness. In contrast, all 30 animals fed the 0.5P diet and reared in 5 mg/L CO_2_ had a typical low-mineralized phenotype with extended areas of non-mineralized bone matrix at the edges of the vertebral bodies and scales. Possible explanations for these curious observations are discussed in the following sections and include (i) a systemic response to respiratory acidosis which likely facilitates increased bone mineralization in animals reared in high CO_2_, (ii) enhanced renal phosphate reabsorption under P-deficiency, (iii) and a reduced P requirement due to reduced growth. 

### 4.2. Can A Systemic Response to Respiratory Acidosis Help Mineralize A Non-Mineralized Bone?

Hypercapnia leads to reduced systemic pH (respiratory acidosis) and increases the level of plasma PCO_2_, bicarbonate ions, and Ca^2+^, while plasma Cl^−^ is reduced [26,93]. Respiratory acidosis compensation is achieved faster and to a higher degree (94–96%) in water with high levels of Ca^2+^ and bicarbonate ions (seawater and hard freshwater) compared with compensation in soft freshwater under low Ca^2+^ conditions (83–88%) [27]. Reduced water pH increases the CaCO_3_ solubility and increases the availability of free Ca^2+^ [13,14]. High ambient bicarbonate ions stimulate an influx of Ca^2+^ via Cl^−^ outflux exchange and further aids in pH stabilization [27]. Animals reared in 20 mg/L CO_2_ in the current study showed a 2.5-fold increase in bicarbonate ions, and 2-fold increase in PCO_2_ and TCO_2_. While the plasma showed a reduced Cl^−^ content, plasma Ca^2+^ increased. The systemic response remarkably led to an increased plasma pH in animals reared in 20 mg/L CO_2_ compared with animals in 5 mg/L CO_2_ water. The pH overcompensation (=high pH) was therefore achieved by the increased influx of Ca^2+^, bicarbonate ions, and outflux of Cl^−^. Such an increased influx of free Ca^2+^ could moreover represent a source of minerals which assisted in the bone mineralization of 0.5P animals reared in 20 mg/L CO_2_. It is possible that the increased Ca^2+^ uptake even increased the intestinal absorption of dietary P. Indeed, the extended areas of non-mineralized bone in 0.5P animals are promptly mineralized when Ca and P become available [38,39,40]. 

### 4.3. Increased Renal Phosphate Reabsorption Driven by the Downregulation of fgf23

Enhanced bone P content can potentially be achieved through increased intestinal absorption from the diet while enhanced renal reabsorption increases phosphate retention. Fibroblast growth factor (*fgf23*), a hormone synthetized by osteoblasts and osteocytes, suppresses renal phosphate reabsorption [89]. The downregulation of *fgf23* in 0.5P animals suggests that the phosphate is retained rather than excreted. Animals fed a 0.5P diet and reared in high CO_2_ showed further downregulation of *fgf23* which suggests enhanced renal phosphate reabsorption. In rainbow trout (*Oncorhynchus mykiss*), deficiency in dietary P increased the expression of sodium-dependent phosphate (Na^+^Pi-IIb) co-transporters responsible for active intestinal phosphate absorption [94,95]. 

### 4.4. Growth Rate and Mineralization

Bone is first laid down as a non-mineralized matrix and only later is it mineralized [96,97,98]. Sustained swimming induces bone mineralization in Atlantic salmon [99,100,101]. In contrast, dietary P exceeding the animals’ requirements has no effect on bone mineralization [39,40,41]. In humans (*Homo sapiens*), the rapid growth of children in puberty results in the development of under-mineralized bone for a transient period of time [102]. Even if the growth is increased, the rate of mineralization remains the same. Mineralization of the fast forming bone matrix during puberty therefore takes a relatively longer time. It is thus expected that minerals are integrated into the bone matrix at a constant rate under regular growth and mechanical load. This indicates that the reduced growth of 0.5P/20CO_2_ animals observed in this study would allow more time for mineral integration into the bone matrix, possibly facilitating bone mineralization even under a P-deficient diet. Comparable to the current study, Gil-Martens et al. [4] observed reduced weight and SGR, and enhanced bone mineral content in animals reared in 35 mg/L CO_2_. Increased bone mineral content in white grouper reared in 28.5 mg/L CO_2_ was associated with a reduced fork length in a study by [45]. Fjelldal et al. [48,73] showed that rapid growth correlates with a reduced mineral content and yield-load, and vertebral centra stiffness in fast growing under-yearling post-smolt Atlantic salmon. 

However, apart from the reduced growth, animals reared in 20 mg/L CO_2_ in the current study also reduced their feed intake. The content of dietary P would thus be equally reduced in animals with a lower appetite. Interestingly, Fivelstad et al. [9] did not record a reduced fork length and weight among Atlantic salmon with increased bone mineral content reared in 16 mg/L or 24 mg/L CO_2_ compared with animals reared in 6 mg/L CO_2_. Thus, the reduced growth is less likely to be responsible for the observed increased bone mineralization in 0.5P/20CO_2_ Atlantic salmon. The differences in vertebra morphology and mineralization between individuals within the same group of animals, as observed in the current 0.5P/20CO_2_ group, have been previously reported in Atlantic salmon reared at presumably ‘friendly’ water CO_2_ levels [43]. In that study, the authors suggested, based on a growth analysis of individuals with different mineralization phenotypes, that reduced appetite in combination with sustained growth in length may cause a low vertebra mineral content in Atlantic salmon post-smolts. In the present study, there is a trend towards an increased mineralization when post-smolts are fed a low P diet and have a reduced appetite due to elevated CO_2_ levels. Taken together, these results concerning growth and mineralization further support the notion that reduced growth alone is not the causal factor for the increased mineralization observed in the 0.5P/20CO_2_ group. This brings up the importance of the previously discussed increased Ca absorption via gill uptake, intestinal Ca, and P absorption, and enhanced renal phosphate reabsorption which argues in favor of the systemic animal response as the main candidate factor for a regular bone mineralization in 0.5P/20CO_2_ animals.

### 4.5. The Negative Effect of High P and CO_2_ on Growth of Animals

High P diet and 20 mg/L of CO_2_ led to the reduction in feed intake and SGR. Reduced feed intake in animals fed a 3P diet is possibly related to the high inclusion of MAP and a high ash content in the diet. Similarly, Sugiura et al. [103] observed that high P diets with high ash content resulted in a reduced feed intake in rainbow trout. Animals reared in 20 mg/L CO_2_ showed a reduced SGR in all diet groups. In studies on European sea bass (*Dicentrarchus labrax*), gilthead seabream (*Sparus aurata*), and rainbow trout, high CO_2_ resulted in olfactory system impairment and reduced SGR [104,105,106,107]. Comparable to other teleost fishes, Atlantic salmon rely on smell (olfactory system) to detect feed [87,88] which could provide an explanation for the reduced feed intake under high CO_2_ in the current study and in other studies [104,105]. In addition, acid-base regulation and stress related to increased plasma CO_2_ are metabolically costly [108,109,110,111]. In accordance with the current findings, it was shown that plasma Cl^−^ levels are significantly reduced in Atlantic salmon smolts exposed to 32 mg/L and 21.8 mg/L CO_2_, compared with animals reared in control conditions (1.7–7.0 mg/L CO_2_) [24,108]. Such a decrease in plasma Cl^−^ is likely associated with the increased bicarbonate levels under high water CO_2_ as a consequence of the HCO_3_^−^/Cl^−^ exchange mechanism [112]. The cumulative effect of impaired smell, the cost of acid-base balance under high CO_2_ condition, stress, and the high ash content of the diet are possible factors for the reduced feed intake and SGR in animals fed the 3P diet and in all diet group animals reared in 20 mg/L CO_2_. 

### 4.6. High Dietary P Does Not Improve Bone Health 

Increased dietary P supplementation in Atlantic salmon diets has been suggested to enhance bone mineralization and prevent vertebral deformities [34,90,91]. Likewise, a diet deficient in dietary P and high water CO_2_ are factors that were suggested to induce vertebral column deformities in Atlantic salmon [30,34,54]. Based on X-ray images, and detailed analyses of Alizarin Red S samples and serial histological sections, the occurrence of mild deformities was equally low in all diet groups. A previously hypothesized association between low P diet, high CO_2_, and the development of deformities is thus not supported by this study. 

Based on various analytical methods (radiography, mineral content analysis, mechanical property analysis, Alizarin Red S staining, non-demineralized histological sections) this study concludes that the increased level of dietary P does not further enhance bone mineralization when compared to recommended levels of dietary P (1P). The analysis of the 3P diet revealed a higher content of Fe (148 mg/kg) relative to 0.5P (78 mg/kg) and 1P (102 mg/kg) diet, comparable to diets used in Drábiková et al. [39] containing on average 163 mg/kg Fe. The likely cause for this increase is a common presence of Fe impurities in MAP [113]. Concentrations of Fe above 60 mg/kg in feed for Atlantic salmon are suggested to reduce vitamin C absorption while dietary Fe oversupply in rodents and in zebrafish were shown to increase bone resorption and reduce bone mineral content [114,115,116]. No adverse effects of Fe content on bone mineralization or bone resorption were observed in the current study nor in the study by Drábiková et al. [39]. Furthermore, the analysis of the expression levels of genes associated with bone mineralization and bone formation (*enpp1*, *entpd5*, *opn*, *col1a1a*, and *bgp*) revealed no differences among the diet groups. The unchanged expression of the bone biomarker genes further confirmed that excessive dietary P had no positive effect on bone formation and bone mineralization. This is in agreement with studies on parr and early-seawater stages of Atlantic salmon where high dietary P intake neither increased bone mineralization nor bone formation [39,40,41].

Under low dietary P conditions in Atlantic salmon and in the zebrafish, formation of the bone matrix is continuous while the bone mineralization is halted [39,40,41,42]. Studies on mineralization of bone in Atlantic salmon with osteomalacia show that the supplementation of P leads to a prompt mineralization of the previously non-mineralized bone matrix [39,40]. This implies that regular bone matrix was formed even under low dietary P conditions and its mineralization was simply delayed by weeks instead of days as is commonly the case [96,97,98]. In line with the previous studies, this study shows a regular bone matrix formation under the 0.5P diet and decoupling of bone formation from bone mineralization. Comparable expressions of gene markers for osteoblasts (*bgp*) and collagen type I (*col1a1a*) in all groups suggest that the bone matrix formation continued regularly in all animals irrespective of P intake. Similarly, the expression of gene markers for inhibition of bone mineralization (*opn* and *enpp1*) and ENTPD5 where the function is to facilitate bone mineralization were not different indicating that proteins required for a proper bone mineralization are synthetized appropriately even under low P conditions. An upregulation of *fgf23* observed in this study as well as in studies by Smedley et al. [54] and Fjelldal et al. [36] indicates increased renal phosphate excretion in animals fed high dietary P. Indeed, several studies provide evidence that a reduced content of dietary P that is within the limits of P requirements, benefits P retention and reduces P waste [117,118,119,120,121,122,123]. A fishmeal-free diet with a reduced content of non-phytin P, supplemented with defluorinated rock phosphate (phosphorus pentoxide), also shows a reduction in solid P waste in juvenile rainbow trout [120]. Rodehutscord et al. [121] determined that rainbow trout fecal P excretion increases gradually with increasing dietary P while non-fecal P excretion increased substantially only after the estimated P requirements are met. In rainbow trout, a high-lipid diet with a lower level of dietary P (315 g lipid, 5.6 g/kg available P) reduces P excretion by nearly 50% and increases P retention by 20% compared with a commercial diet (173 g lipid, 6.9 g/kg available P) [117]. These studies and the current findings demonstrate benefits of reduced dietary P inclusion concerning reduced P effluents from fish farms without compromising the animals’ bone health. The current study is especially interesting in the context of emerging aquaculture needs for use of higher stocking densities and recirculating systems, both of which are inherently associated with increased CO_2_ release into the water [1,2,6]. Other studies have already elaborated on increased bone mineral content in animals reared in a high CO_2_ environment [1,4,9,44,45]. Here it is indicated that the requirement for dietary P in seawater Atlantic salmon is reduced when reared in water with 20 mg/L CO_2_. A study under regular commercial farming conditions could show if the reduction in dietary P in the Atlantic salmon diet is possible for fish reared in recirculating systems or high stocking densities.

## 5. Conclusions

This study analyzed the synergistic effect of high water CO_2_ (20 mg/L CO_2_) and low or high dietary P on bone formation and bone mineralization in post-smolt Atlantic salmon. High dietary P (3P) had no further effect on bone mineralization and bone matrix formation in post-smolt Atlantic salmon. High dietary P (3P) and 20 mg/L CO_2_ had detrimental effects on SGR, arguably as a result of the decreased feed intake. Animals fed low dietary P (0.5P) and reared in 20 mg/L CO_2_ either showed bone mineralization comparable to Atlantic salmon fed the recommended (1P) and high (3P) levels of dietary P. However, some animals from the 0.5P/20CO_2_ developed a mild low-mineralized phenotype. This phenotype was, nevertheless, less severe than the phenotype in the Atlantic salmon 0.5P/5CO_2_ group. The positive effect of high water CO_2_ on bone mineralization coincided with the downregulation of *fgf23* expression which suggests that increased renal phosphate reabsorption is responsible for maintaining normal levels of mineralization. The possible reduction in dietary P requirement offers an opportunity to lower the P content in feeds for salmon reared in systems in which CO_2_ accumulates. These include land-based systems with high stocking densities and recirculating systems.

## Figures and Tables

**Table 1:** Formulation and analyzed composition of experimental diets.

Diet	0.5P	1P	3P
Formulation (g/kg)			
Wheat gluten	241.8	243.6	243.7
Fishmeal ^ab^	250.0	250.0	250.0
Tapioca Starch 79.5%	71.5	72.0	71.3
Sodium caseinate 89.2%	61.0	59.4	59.4
Guar meal 58%	9.8	9.8	9.7
Wheat	31.3	31.3	31.2
Mono-ammonium phosphate ^a^	0.0	11.9	88.2
Cellulose	92.2	80.5	0.0
Fish oil ^c^	78.9	78.4	78.5
Rapeseed oil	150.0	149.0	149.1
Premixes ^d^	15.8	15.9	15.9
Water/ Moisture change ^e^	−2.3	−1.8	2.9
Proximate analysis			
Moisture (%)	6.1	6.8	6.3
Crude protein (%)	43.4	43.7	49.5
Crude fat (%)	26.9	26.1	27.5
Ash (%)	4.4	4.8	9.4
Total P g/kg	6.3	9.0	26.8
Estimated available P g/kg ^f^	2.8	5.6	23.4
Total Fe, mg/kg	78.0	102.0	148.0
Astaxanthin mg/kg	63.0	60.5	58.9

^a^ Skretting; ^b^ North Atlantic (Skretting); ^c^ Northern hemisphere oil (Skretting); ^d^ Include vitamins and minerals (Trouw Nutrition) and amino acids (Skretting), estimated to cover requirements of Atlantic salmon according to the National Research Council (2011); ^e^ Water added during feed processing to adjust for different moisture content of the raw materials in order to meet targeted moisture content in the final feed; ^f^ Calculated based on content of total P, digestible P of raw material given in National Research Council [60], and digestible P of mono-ammonium phosphate in Morales et al. [50].

**Table 2:** Primer pairs used to amplify reference genes and genes of interest in real time PCR.

Target	Primer Sequence 5′ to 3′	Accession Number	Amplicon Size (bp*)	Annealing Temperature	Reference
*β-actin*	F: CAACTGGGACGACATGGAGA R: AGTGAGCAGGACTGGGTGCT	AF012125	88	58	[80]
*ef1a*	F: GCACCACGAGACCCTGGAAT R: CACGTTGCCACGACGATAT	AF321836	94	60	[79]
*bgp*	F: GACTCCTCTACCTCCACTGC R: AATGATCCCAGCTGTGTCCA	NM_001136551.1	207	60	[77]
*col1a1a*	F: TGGTGAGCGTGGTGAGTCTG R: TAGCTCCGGTGTTTCCAGCG	FJI95608	188	60	[77]
*opn*	F: CTTACTGAGGTGGCCCCTGT R: GCTGTCCGATGTTGGGTCTG	XM_014186048.1	114	60	[77]
*enpp1*	F: ACGGACCAATGAGCAGTCAG R: TGAGTTCACGAATGCAGCCT	XM_014204668.1	173	60	Own design
*entpd5*	F: AGCCGTACGAGATAAAGGGC R: CCCCGACTGCCATCAATGAG	XM_0141843607.2	94	60	Own design
*fgf23*	F: ACTGGGGGAATCCGAGAAGA R: TCTTCCTTGTGGCAAACGGT	XM_014153467.2	241	60	Own design

* bp—base pairs; *ef1a*—elongation factor 1 alpha; *bgp*—bone gla protein; *col1a1a*—collagen type 1 alpha 1a; *opn*—osteopontin; *enpp1*—ectonucleotide pyrophosphate/phosphodi-esterase 1; *entpd 5*—ectonucleoside triphosphate diphosphohydrolase 5; *fgf 23*—fibroblast growth factor 23.

**Table 3:** Growth data of Atlantic salmon measured at weeks 3, 8, and 16. Animals were fed diets containing different levels of total P 0.5P (6.3 g/kg), 1P (9.0 g/kg), and 3P (26.8 g/kg) and reared in conditions with no CO_2_ injected (5 mg/L) or with injected CO_2_ (20 mg/L) from week 3 till week 16. The statistically significant differences among groups (within a row) are indicated by different lowercase superscript letters. For SGR, FCR, feed intake, and total feed eaten, tanks were considered as statistical replicates and variances among groups were therefore not analyzed. If none of the letters are the same, differences are significant. The significant values (*p*) for CO_2_, diet (D), and their interaction are indicated.

CO_2_	5 mg/L CO_2_			20 mg/L CO_2_			*p*-Value		
**Diet/** **Measurements**	**0.5P**	**1P**	**3P**	**0.5P**	**1P**	**3P**	**D**	**CO**_**2**_	**D × CO**_**2**_
Week 3									
**Fork length (cm)**	25.97 ± 0.85	26.04 ± 0.89	25.81 ± 0.78	26.00 ± 0.91	26.25 ± 1.09	25.82 ± 0.85	- ^1^	-	-
**Weight (g)**	204. 28 ± 18.84	205. 15 ± 21.73	205.14 ± 21.87	204.60 ± 18.01	207.22 ± 21.45	207.02 ± 19.35	-	-	-
**Condition factor**	1.18 ± 0.07	1.20 ± 0.08	1.20 ± 0.05	1.18 ± 0.08	1.15 ± 0.06	1.18 ± 0.06	-	-	-
Week 8									
**Fork length (cm)**	29.33 ± 1.01 ^ab^	29.97 ± 1.47 ^b^	29.17 ± 1.12 ^ab^	28.50 ± 1.34 ^a^	28.72 ± 1.28 ^a^	29.22 ± 1.22 ^ab^	0.245	0.250	**0.026**
**Weight (g)**	299.87 ± 30.19 ^ab^	326.40 ± 48.04 ^b^	287.53 ± 35.37 ^a^	269.60 ± 42.40 ^a^	270.87 ± 43.50 ^a^	284.87 ± 38.96 ^a^	0.117	**<0.001**	**0.002**
**Condition factor**	1.19 ± 0.09 ^ab^	1.21 ± 0.09 ^b^	1.16 ± 0.07 ^ab^	1.16 ± 0.07 ^ab^	1.14 ± 0.08 ^a^	1.14 ± 0.06 ^a^	0.059	**0.002**	0.608
Week 16									
**Fork length (cm)**	36.02 ± 2.02 ^bc^	36.92 ± 1.56 ^c^	35.67 ± 2.00 ^abc^	34.30 ± 1.53 ^a^	35.49 ± 2.07 ^ab^	35.24 ± 1.55 ^a^	**0.009**	**<0.001**	0.056
**Weight (g)**	586.47 ± 79.47 ^bc^	635.13 ± 96.50 ^c^	531.33 ± 105.90 ^ab^	484.33 ± 79.87 ^a^	526.13 ± 85.42 ^ab^	527.80 ± 66.09 ^ab^	**0.002**	**<0.001**	**0.001**
**Condition factor**	1.26 ± 0.21 ^ab^	1.25 ± 0.10 ^b^	1.16 ± 0.15 ^ab^	1.19 ± 0.08 ^ab^	1.17 ± 0.07 ^a^	1.20 ± 0.07 ^ab^	0.192	0.059	**0.002**
**SGR ^2^** **(% body mass day** ^**−1**^ **) ** ^**3**^	1.17 ± 0.01	1.25 ± 0.05	1.10 ± 0.01	0.99 ± 0.02	1.10 ± 0.06	1.06 ± 0.01	**<0.001**	**0.010**	0.056
**FCR ^4^ (DM ^5^) ^ 3^**	0.71 ± 0.00	0.69 ± 0.01	0.70 ± 0.00	0.75 ± 0.00	0.72 ± 0.01	0.73 ± 0.00	**<0.001**	**<0.001**	0.847
**Feed intake (% of body mass day** ^**−1**^ **) ** ^**3**^	0.89 ± 0.01	0.92 ± 0.03	0.82 ± 0.01	0.79 ± 0.02	0.85 ± 0.05	0.82 ± 0.00	**0.010**	**0.027**	0.064
**Total feed eaten (kg DM) ^3^**	10.28 ± 0.00	11.12 ± 0.32	8.82 ± 0.04	8.17 ± 0.57	8.81 ± 0.61	8.18 ± 0.01	**<0.001**	**0.004**	0.034

^1^ Two-way ANOVA was not tested on initial sampled; ^2^ Specific growth rate; ^3^ Specific differences between groups were not determined; ^4^ Feed conversion ratio; ^5^ Dry matter.

**Table 5:** Extent of non-mineralized bone in zygapophyses. The analysis was performed on whole mount Alizarin Red S stained vertebral columns of animals fed diets containing different levels of total P 0.5P (6.3 g/kg), 1P (9.0 g/kg), and 3P (26.8 g/kg) and reared in conditions with no CO_2_ injected (5 mg/L) or with injected CO_2_ (20 mg/L). The extent of non-mineralized bone on both pre-zygapophyses and post-zygapophyses was significantly increased in 0.5P/5CO_2_ animals. The statistically significant differences among groups (within a row) are indicated by different lowercase superscript letters. If none of the letters are the same, differences are significant. The significant values (*p*) for CO_2_, diet (D), and their interaction are indicated.

CO_2_	5 mg/L CO_2_			20 mg/L CO_2_			*p*-Value		
**Diet/Measurements**	**0.5P**	**1P**	**3P**	**0.5P**	**1P**	**3P**	**D**	**CO**_**2**_	**D × CO**_**2**_
**Pre-zygapophyses (mm)**	0.13 ± 0.09 ^b^	0.02 ± 0.00 ^a^	0.02 ± 0.00 ^a^	0.07 ± 0.03 ^ab^	0.02 ± 0.01 ^a^	0.02 ± 0.00 ^a^	**<0.001**	0.113	0.123
**Post-zygapophyses (mm)**	0.12 ± 0.05 ^b^	0.03 ± 0.01 ^bc^	0.02 ± 0.01 ^bc^	0.08 ± 0.02 ^ab^	0.04 ± 0.01 ^b^	0.02 ± 0.00 ^bc^	**<0.001**	0.286	0.078

**Table 6:** Whole blood analysis (iSTAT). Listed values for pH, PCO_2_ (partial pressure of CO_2_), bicarbonate ions, TCO_2_ (total CO_2_), PO_2_ (partial pressure of O_2_), and sO_2_ (O_2_ saturation) of Atlantic salmon at weeks 3 and 8 fed different levels of total phosphorus (P) 0.5P (6.3 g/kg), 1P (9.0 g/kg), and 3P (26.8 g/kg) and reared in conditions with no CO_2_ injected (5 mg/L) or with injected CO_2_ (20 mg/L). The statistically significant differences among groups (within a row) are indicated by different lowercase superscript letters. If none of the letters are the same, differences are significant. The significant values (*p*) for CO_2_, diet (D), and their interaction are indicated.

CO_2_		5 mg/L			20 mg/L			*p*-Value		
	Week 3	Week 16								
**Diet/Measurements**	**0.5P**	**0.5P**	**1P**	**3P**	**0.5P**	**1P**	**3P**	**D**	**CO**_**2**_	**D × CO**_**2**_
**pH**	7.06 ± 0.02	6.99 ± 0.04 ^b^	7.02 ± 0.04 ^b^	7.03 ± 0.05 ^b^	7.20 ± 0.05 ^c^	7.17 ± 0.05 ^c^	7.17 ± 0.03 ^c^	0.993	**<0.001**	0.092
**PCO_2_ (kPa)**	5.25 ± 0.45	3.80 ± 0.45 ^a^	3.59 ± 0.35 ^a^	3.62 ± 0.44 ^a^	6.43 ± 0.87 ^b^	7.12 ± 0.57 ^b^	7.28 ± 0.72 ^b^	0.226	**<0.001**	**0.018**
**Bicarbonate ions (mmol/L)**	11.08 ± 0.60	6.93 ± 0.62 ^a^	6.88 ± 0.40 ^a^	7.05 ± 0.32 ^a^	18.94 ± 0.81 ^b^	19.58 ± 1.29 ^b^	19.93 ± 0.66 ^b^	0.098	**<0.001**	0.178
**TCO_2_ (mmol/L)**	12.33 ± 0.52	7.88 ± 0.75 ^a^	7.70 ± 0.50 ^a^	7.90 ± 0.22 ^a^	20.33 ± 1.02 ^b^	21.20 ± 1.24 ^b^	21.60 ± 0.72 ^b^	0.076	**<0.001**	0.053
**PO_2_ (kPa)**	4.98 ± 2.48	4.95 ± 1.06	5.33 ± 1.11	4.89 ± 1.56	5.72 ± 0.91	5.68 ± 1.75	4.57 ± 0.72	0.167	0.475	0.432
**sO_2_ (%)**	45.17 ± 24.72	44.28 ± 14.79 ^ab^	30.63 ± 13.69 ^a^	45.55 ± 21.34 ^ab^	66.18 ± 11.67 ^b^	61.10 ± 20.71 ^b^	49.80 ± 10.01 ^ab^	0.259	**0.009**	0.280

**Table 7:** Blood plasma analysis (Konelab). Listed values for different plasma alkaline phosphatase (ALP), bone/liver/kidney ALP (b/l/k ALP), pH, bicarbonate ions, total and non-bound Ca^2+^, P*i*, Cl^−^, and Zn^2+^ of Atlantic salmon at weeks 3 and 16 fed different levels of total phosphorus (P) 0.5P (6.3 g/kg), 1P (9.0 g/kg), and 3P (26.8 g/kg) and reared in conditions with no CO_2_ injected (5 mg/L) or with injected CO_2_ (20 mg/L). The statistically significant differences among groups (within a row) are indicated by different lowercase superscript letters. If none of the letters are the same, differences are significant. The significant values (*p*) for CO_2_, diet (D), and their interaction are indicated.

CO_2_		5 mg/L			20 mg/L			*p*-Value		
	Week 3	Week 16								
**Diet/** **Measurements**	**0.5P**	**0.5P**	**1P**	**3P**	**0.5P**	**1P**	**3P**	**D**	**CO_2_**	**D × CO_2_**
**ALP (U/L)**	161.00 ± 32.14	158.63 ± 37.62 ^a^	196.98 ± 45.61 ^ab^	217.00 ± 46.93 ^ab^	184.33 ± 33.59 ^ab^	232.61 ± 35.63 ^ab^	285.26 ± 75.23 ^b^	**0.001**	**0.011**	0.530
**b/l/k ALP (U/L)**	-	103.35 ± 28.50 ^a^	126.93 ± 37.78 ^ab^	139.74 ± 34.74 ^ab^	119.58 ± 25.43 ^ab^	152.18 ± 29.11 ^ab^	188.23 ± 61.49 ^b^	**0.008**	**0.025**	0.564
**pH**	7.20 ± 0.08	7.20 ± 0.07 ^a^	7.14 ± 0.09 ^a^	7.08 ± 0.16 ^a^	7.39 ± 0.09 ^b^	7.40 ± 0.09 ^b^	7.37 ± 0.13 ^b^	**0.020**	**<0.001**	0.173
**Bicarbonate ions (mmol/L)**	10.60 ± 1.18	10.76 ± 1.42 ^a^	10.10 ± 1.32 ^a^	10.11 ± 1.49 ^a^	23.34 ± 1.89 ^b^	23.39 ± 1.98 ^b^	24.30 ± 1.92 ^b^	0.482	**<0.001**	0.124
**Total Ca^2+^ (mmol/L)**	3.50 ± 0.34	3.14 ± 0.14 ^a^	3.29 ± 0.13 ^ab^	3.30 ± 0.32 ^ab^	3.28 ± 0.17 ^ab^	3.52 ± 0.17^c^	3.57 ± 0.13 ^c^	**<0.001**	**<0.001**	0.288
**Non-bound Ca^2+^ (mmol/L)**	1.8 ± 0.11	1.46 ± 0.06 ^abc^	1.43 ± 0.07 ^ab^	1.45 ± 0.11 ^ab^	1.52 ± 0.07 ^cd^	1.54 ± 0.04 ^d^	1.54 ± 0.07 ^d^	**0.020**	**<0.001**	0.419
**P*i* (mmol/L)**	3.40 ± 0.62	2.19 ± 0.22 ^a^	3.85 ± 0.36 ^b^	3.56 ± 0.31 ^b^	2.26 ± 0.51 ^a^	3.73 ± 0.25 ^b^	3.69 ± 0.25 ^b^	**<0.001**	0.671	0.231
**Cl^−^ (mmol/L)**	108.50 ± 2.07	116.45 ± 1.70 ^b^	115.65 ± 1.63 ^b^	115.15 ± 2.72 ^b^	112.50 ± 3.28 ^ab^	111.10 ± 1.41 ^a^	111.70 ± 1.45 ^a^	**0.039**	**<0.001**	0.522
**Zn^2+^ (mmol/L)**	68.60 ± 15.40	215.35 ± 41.07 ^c^	205.4 ± 30.99 ^bc^	172.80 ± 34.80 ^ab^	180.80 ± 28.77 ^abc^	165.00 ± 23.06 ^a^	155.85 ± 31.34 ^a^	**<0.001**	**<0.001**	0.222

**Table 8:** Mechanical properties of vertebral centra. Analyzed mechanical properties were corrected for size of the vertebral centra. Three animals per tank (six/group) were measured at week 16. Animals were fed different levels of total phosphorus (P) 0.5P (6.3 g/kg), 1P (9.0 g/kg), and 3P (26.8 g/kg) and reared in conditions with no CO_2_ injected (5 mg/L) or with injected CO_2_ (20 mg/L). The statistically significant differences among groups (within a row) are indicated by different lowercase superscript letters. If none of the letters are the same, differences are significant. The significant values (*p*) for CO_2_, diet (D), and their interaction are indicated.

CO_2_	5 mg/L CO_2_			20 mg/L CO_2_			*p*-Value		
**Diet/** **Measurements**	**0.5P**	**1P**	**3P**	**0.5P**	**1P**	**3P**	**D**	**CO_2_**	**D × CO_2_**
**Modulus of Elasticity**	0.15 ± 0.04 ^a^	0.60 ± 0.12 ^b^	0.71 ± 0.12 ^b^	0.25 ± 0.07 ^a^	0.70 ± 0.10 ^b^	0.65 ± 0.12 ^b^	**<0.001**	0.152	**0.012**
**Yield point (0.2% offset)**	1.56 ± 0.31 ^a^	3.18 ± 0.89 ^bc^	3.30 ± 0.82 ^c^	2.39 ± 0.44 ^ab^	3.75 ± 0.79 ^c^	3.27 ± 0.75 ^bc^	**<0.001**	**0.023**	0.103
**Failure point (5% offset)**	2.40 ± 0.27 ^a^	4.92 ± 0.86 ^b^	5.55 ± 0.44 ^b^	3.17 ± 0.30 ^a^	5.29 ± 0.68 ^b^	5.19 ± 0.38 ^b^	**<0.001**	0.165	0.039
**Toughness** **(MPa x%)**	31.46 ± 5.79 ^a^	43.77 ± 10.38 ^ab^	47.45 ± 8.32 ^b^	36.60 ± 4.87 ^ab^	46.01 ± 8.23 ^ab^	46.54 ± 9.93 ^b^	**<0.001**	0.377	0.736

## Data Availability

The data presented in this study are available on request from the corresponding author.

## References

[1] Helland S., Refstie S., Espmark Å., Hjelde K., Baeverfjord G. (2005). Mineral balance and bone formation in fast-growing Atlantic salmon parr (*Salmo salar*) in response to dissolved metabolic carbon dioxide and restricted dietary phosphorus supply. Aquaculture.

[2] Skov P.V., Grosell M., Munday P.L., Farrell A.P., Brauner C.J. (2019). CO_2_ in aquaculture. Carbon Dioxide.

[3] Bergheim A., Fivelstad S., Woo P.T.K., Noakes D.J. (2014). Atlantic salmon (*Salmo salar* L.) in aquaculture: Metabolic rate and water flow requirements. Salmon: Biology, Ecological Impacts and Economical Importance.

[4] Gil-Martens L., Witten P.E., Fivelstad S., Huysseune A., Sævareid B., Vikeså V., Obach A. (2006). Impact of high water carbon dioxide levels on Atlantic salmon smolts (*Salmo salar* L.): Effects on fish performance, vertebrae composition and structure. Aquaculture.

[5] Good C., Davidson J. (2016). A review of factors influencing maturation of Atlantic salmon, *Salmo salar*, with focus on water recirculation aquaculture system environments. J. World Aquac. Soc..

[6] Good C., Davidson J., Terjesen B.F., Takle H., Kolarevic J., Bæverfjord G., Summerfelt S. (2018). The effects of long-term 20 mg/L carbon dioxide exposure on the health and performance of Atlantic salmon *Salmo salar* post-smolts in water recirculation aquaculture systems. Aquac. Eng..

[7] Crouse C., Davidson J., May T., Summerfelt S., Good C. (2021). Production of market-size European strain Atlantic salmon (*Salmo salar*) in land-based freshwater closed containment aquaculture systems. Aquac. Eng..

[8] Fivelstad S., Haavik H., Løvik G., Olsen A.B. (1998). Sublethal effects and safe levels of carbon dioxide in seawater for Atlantic salmon postsmolts (*Salmo salar* L.): Ion regulation and growth. Aquaculture.

[9] Fivelstad S., Olsen A.B., Åsgård T., Baeverfjord G., Rasmussen T., Vindheim T., Stefansson S. (2003). Long-term sublethal effects of carbon dioxide on Atlantic salmon smolts (*Salmo salar* L.): Ion regulation, haematology, element composition, nephrocalcinosis and growth parameters. Aquaculture.

[10] Mota V.C., Nilsen T.O., Gerwins J., Gallo M., Kolarevic J., Krasnov A., Terjesen B.F. (2020). Molecular and physiological responses to long-term carbon dioxide exposure in Atlantic salmon (*Salmo salar*). Aquaculture.

[11] Wilson R.W., Millero F.J., Taylor J.R., Walsh P.J., Christensen V., Jennings S., Grosell M. (2009). Contribution of fish to the marine inorganic carbon cycle. Science.

[12] McNeil B.I., Matsumoto K., Grosell M., Munday P.L., Farrell A.P., Brauner C.J. (2019). The changing ocean and freshwater CO_2_ system. Carbon Dioxide.

[13] Goss S.L., Lemons K.A., Kerstetter J.E., Bogner R.H. (2007). Determination of calcium salt solubility with changes in pH and PCO_2_, simulating varying gastrointestinal environments. J. Pharm. Pharmacol..

[14] Hofmann L., Bischof K. (2014). Ocean acidification effects on calcifying macroalgae. Aquat. Biol..

[15] Perry S.F. (1982). The regulation of hypercapnic acidosis in two Salmonids, the freshwater trout (*Salmo gairdneri*) and the seawater salmon (*Onchorynchus kisutch*). Mar. Behav. Physiol..

[16] Toews D.P., Holeton G.F., Heisler N. (1983). Regulation of the acid-base status during environmental hypercapnia in the marine teleost fish *Conger conger*. J. Exp. Biol..

[17] Munday P.L., Rummer J.L., Baumann H., Grosell M., Munday P.L., Farrell A.P., Brauner C.J. (2019). Adaptation and evolutionary responses to high CO_2_. Carbon Dioxide.

[18] Mortimer C.H. (1956). The oxygen content of air-saturated fresh waters, and aids in calculating percentage saturation. Int. Ver. Theor. Angewadte Limnol. Mitt..

[19] Domenici P., Steffensen J.F., Marras S. (2017). The effect of hypoxia on fish schooling. Phil. Trans. R. Soc. B.

[20] Rummer J.L., McKenzie D.J., Innocenti A., Supuran C.T., Brauner C.J. (2013). Root effect hemoglobin may have evolved to enhance general tissue oxygen delivery. Science.

[21] Eddy F., Lomholt J., Weber R., Johansen K. (1977). Blood respiratory properties of rainbow trout (*Salmo gairdneri*) kept in water of high CO_2_ tension. J. Exp. Biol..

[22] Fivelstad S., Hosfeld C.D., Medhus R.A., Olsen A.B., Kvamme K. (2018). Growth and nephrocalcinosis for Atlantic salmon (*Salmo salar* L.) post-smolt exposed to elevated carbon dioxide partial pressures. Aquaculture.

[23] Fivelstad S., Kvamme K., Handeland S., Fivelstad M., Olsen A.B., Hosfeld C.D. (2015). Growth and physiological models for Atlantic salmon (*Salmo salar* L.) parr exposed to elevated carbon dioxide concentrations at high temperature. Aquaculture.

[24] Fivelstad S., Olsen A.B., Kloften H., Ski H., Stefansson S. (1999). Effects of carbon dioxide on Atlantic salmon (*Salmo salar* L.) smolts at constant pH in bicarbonate rich freshwater. Aquaculture.

[25] Fivelstad S., Waagbø R., Stefansson S., Olsen A.B. (2007). Impacts of elevated water carbon dioxide partial pressure at two temperatures on Atlantic salmon (*Salmo salar* L.) parr growth and haematology. Aquaculture.

[26] Mota V.C., Nilsen T.O., Gerwins J., Gallo M., Ytteborg E., Baeverfjord G., Kolarevic J., Summerfelt S.T., Terjesen B.F. (2019). The effects of carbon dioxide on growth performance, welfare, and health of Atlantic salmon post-smolt (*Salmo salar*) in recirculating aquaculture systems. Aquaculture.

[27] Larsen B.K., Jensen F.B. (1997). Influence of ionic composition on acid-base regulation in rainbow trout (*Oncorhynchus mykiss*) exposed to environmental hypercapnia. Fish Physiol. Biochem..

[28] Sullivan M., Guy D.R., Roberts R.J., Manchester N.J. (2007). The aetiology of spinal deformity in Atlantic salmon, *Salmo salar* L.: Influence of genetic factors on the frequency and severity in freshwater stages. J. Fish Dis..

[29] Lin C.-H., Hwang P.-P. (2016). The control of calcium metabolism in zebrafish (*Danio rerio*). Int. J. Mol. Sci..

[30] Al-Kholy A., Ishak M.M., Youssef Y.A., Khalil S.R. (1970). Phosphorus uptake from water by *Tilapia zillii* (Gervais). Hydrobiologia.

[31] Llorens C.P. (2022). Differential Phosphorus Uptake by Juvenile European Catfish (*Silurus glanis*) from Feed and Water in Recirculating Aquaculture System. Master’s Thesis.

[32] van Bussel C.G.J., Mahlmann L., Kroeckel S., Schroeder J.P., Schulz C. (2013). The effect of high ortho-phosphate water levels on growth, feed intake, nutrient utilization and health status of juvenile turbot (*Psetta maxima*) reared in intensive recirculating aquaculture systems (RAS). Aquac. Eng..

[33] Lall S., Kaushik S. (2021). Nutrition and metabolism of minerals in fish. Animals.

[34] Baeverfjord G., Åsgård T., Shearer K.D. (1998). Development and detection of phosphorus deficiency in Atlantic salmon, *Salmo salar* L.; parr and post-smolts. Aquac. Nutr..

[35] Fjelldal P.G., Hansen T., Breck O., Sandvik R., WaagbØ R., Berg A., Ørnsrud R. (2009). Supplementation of dietary minerals during the early seawater phase increase vertebral strength and reduce the prevalence of vertebral deformities in fast-growing under-yearling Atlantic salmon (*Salmo salar* L.) smolt. Aquac. Nutr..

[36] Fjelldal P.G., Hansen T.J., Lock E.J., Wargelius A., Fraser T.W.K., Sambraus F., El-Mowafi A., Albrektsen S., Waagbø R., Ørnsrud R. (2016). Increased dietary phosphorous prevents vertebral deformities in triploid Atlantic salmon (*Salmo salar* L.). Aquac. Nutr..

[37] Storebakken T., Shearer K., Roem A. (2000). Growth, uptake and retention of nitrogen and phosphorus, and absorption of other minerals in Atlantic salmon *Salmo salar* fed diets with fish meal and soy-protein concentrate as the main sources of protein. Aquac. Nutr..

[38] Drábiková L., Fjelldal P.G., De Clercq A., Yousaf M.N., Morken T., McGurk C., Witten P.E. (2022). What will happen to my smolt at harvest? Individually tagged Atlantic salmon help to understand possible progression and regression of vertebral deformities. Aquaculture.

[39] Drábiková L., Fjelldal P.G., De Clercq A., Yousaf M.N., Morken T., McGurk C., Witten P.E. (2021). Vertebral column adaptations in juvenile Atlantic salmon *Salmo salar*, L. as a response to dietary phosphorus. Aquaculture.

[40] Witten P.E., Fjelldal P.G., Huysseune A., McGurk C., Obach A., Owen M.A.G. (2019). Bone without minerals and its secondary mineralization in Atlantic salmon (*Salmo salar*): The recovery from phosphorus deficiency. J. Exp. Biol..

[41] Witten P.E., Owen M.A.G., Fontanillas R., Soenens M., Mcgurk C., Obach A. (2016). A primary phosphorus-deficient skeletal phenotype in juvenile Atlantic salmon *Salmo salar*: The uncoupling of bone formation and mineralization. J. Fish Biol..

[42] Cotti S., Huysseune A., Koppe W., Rücklin M., Marone F., Wölfel E., Fiedler I., Busse B., Forlino A., Witten P. (2020). More bone with less minerals? The effects of dietary phosphorus on the post-cranial skeleton in zebrafish. Int. J. Mol. Sci..

[43] Fjelldal P.G., Nordgarden U., Hansen T. (2007). The mineral content affects vertebral morphology in underyearling smolt of Atlantic salmon (*Salmo salar* L.). Aquaculture.

[44] Canzanello V.J., Kraut J.A., Holick M.F., Johns C., Liu C.C., Madias N.E. (1995). Effect of chronic respiratory acidosis on calcium metabolism in the rat. J. Lab. Clin. Med..

[45] Elsadin S., Nixon O., Mozes N., Allon G., Gaon A., Tandler A., Koven W. (2019). The effect of dissolved carbon dioxide (CO_2_) on the bone mineral content and on the expression of bone-Gla protein (BGP, Osteocalcin) in the vertebral column of white grouper (*Epinephelus aeneus*). Aquaculture.

[46] Smart G.R., Knox D., Harrison J.G., Ralph J.A., Richards R.H., Cowey C.B. (1979). Nephrocalcinosis in rainbow trout *Salmo gairdneri* Richardson; the effect of exposure to elevated CO_2_ concentrations. J. Fish Dis..

[47] Björnsson B.T., Hemre G.-I., Bjørnevik M., Hansen T. (2000). Photoperiod regulation of plasma growth hormone levels during induced smoltification of underyearling Atlantic salmon. Gen. Comp. Endocrinol..

[48] Fjelldal P.G., Lock E.-J., Grotmol S., Totland G.K., Nordgarden U., Flik G., Hansen T. (2006). Impact of smolt production strategy on vertebral growth and mineralisation during smoltification and the early seawater phase in Atlantic salmon (*Salmo salar*, L.). Aquaculture.

[49] Hossain M.S., Chance A.B., El Kertaoui N., Wattiez X., Houndji A., Mandiki S.N.M., Kestemont P. (2020). Dietary inorganic monophosphates in high plant ingredient-based diets influence nutrient digestibility, postprandial macro-mineral status and immune functions of juvenile rainbow trout, *Oncorhynchus mykiss*. Aquacult. Nutr..

[50] Morales G.A., Azcuy R.L., Casaretto M.E., Márquez L., Hernández A.J., Gómez F., Koppe W., Mereu A. (2018). Effect of different inorganic phosphorus sources on growth performance, digestibility, retention efficiency and discharge of nutrients in rainbow trout (*Oncorhynchus mykiss*). Aquaculture.

[51] Milián-Sorribes M.C., Tomás-Vidal A., Peñaranda D., Carpintero L., Sebastian J., Dupuy Arnau J., Donadeu A., Macías-Vidal J., Martínez-Llorens S. (2021). Estimation of phosphorus and nitrogen waste in rainbow trout (*Oncorhynchus mykiss*, Walbaum, 1792) Diets including different inorganic phosphorus sources. Animals.

[52] Albrektsen S., Lock E.J., Bæverfjord G., Pedersen M., Krasnov A., Takle H., Veiseth-Kent E., Ørnsrud R., Waagbø R., Ytteborg E. (2018). Utilization of H2SO4-hydrolysed phosphorus from herring bone by-products in feed for Atlantic salmon (*Salmo salar*) 0+postsmolt. Aquac. Nutr..

[53] Fjelldal P.G., Hansen T., Albrektsen S. (2012). Inadequate phosphorus nutrition in juvenile Atlantic salmon has a negative effect on long-term bone health. Aquaculture.

[54] Smedley M.A., Migaud H., McStay E.L., Clarkson M., Bozzolla P., Campbell P., Taylor J.F. (2018). Impact of dietary phosphorous in diploid and triploid Atlantic salmon (*Salmo salar* L.) with reference to early skeletal development in freshwater. Aquaculture.

[55] Krogdahl Å., Hemre G.I., Mommsen T.O. (2005). Carbohydrates in fish nutrition: Digestion and absorption in postlarval stages. Aquacult. Nutr..

[56] Bergot F. (1981). Digestibility of a purified cellulose by the rainbow trout (*Salmo gairdnerii*) and the common carp (*Cyprinus carpio*). Reprod. Nutr. Develop..

[57] Kraugerud O.F., Penn M., Storebakken T., Refstie S., Krogdahl Å., Svihus B. (2007). Nutrient digestibilities and gut function in Atlantic salmon (*Salmo salar*) fed diets with cellulose or non-starch polysaccharides from soy. Aquaculture.

[58] Hansen J.Ø., Storebakken T. (2007). Effects of dietary cellulose level on pellet quality and nutrient digestibilities in rainbow trout (*Oncorhynchus mykiss*). Aquaculture.

[59] Bromley P.J., Adkins T.C. (1984). The influence of cellulose filler on feeding, growth and utilization of protein and energy in rainbow trout, *Salmo gairdnerii* Richardson. J. Fish Biol..

[60] Busacker G.P., Adelman I.R., Goolish E.M., Schreck C.B., Moyle P.B. (1990). Growth. Methods for Fish Biology.

[61] National Research Council (2011). Nutrient Requirements of Fish and Shrimp.

[62] Ponder B.A., Wilkinson M.M. (1981). Inhibition of endogenous tissue alkaline phosphatase with the use of alkaline phosphatase conjugates in immunohistochemistry. J. Histochem. Cytochem..

[63] Witten P.E. (1997). Enzyme histochemical characteristics of osteoblasts and mononucleated osteoclasts in a teleost fish with acellular bone (*Oreochromis niloticus*, Cichlidae). Cell Tissue Res..

[64] De Clercq A., Perrott M.R., Davie P.S., Preece M.A., Wybourne B., Ruff N., Huysseune A., Witten P.E. (2017). Vertebral column regionalisation in Chinook salmon, *Oncorhynchus tshawytscha*. J. Anat..

[65] Witten P.E., Gil-Martens L., Huysseune A., Takle H., Hjelde K. (2009). Towards a classification and an understanding of developmental relationships of vertebral body malformations in Atlantic salmon (*Salmo salar* L.). Aquaculture.

[66] Arratia G., Schultze H.-P. (1992). Reevaluation of the caudal skeleton of certain actinopterygian fishes: III. Salmonidae. Homologization of caudal skeletal structures. J. Morphol..

[67] Arratia G., Schultze H.P., Casciotta J. (2001). Vertebral column and associated elements in dipnoans and comparison with other fishes: Development and homology. J. Morphol..

[68] Britz R., Johnson G.D. (2005). Occipito-vertebral fusion in ocean sunfishes (Teleostei: Tetraodontiformes: Molidae) and its phylogenetic implications. J. Morphol..

[69] De Clercq A., Perrott M.R., Davie P.S., Preece M.A., Owen M.A.G., Huysseune A., Witten P.E. (2018). Temperature sensitive regions of the *Chinook salmon* vertebral column: Vestiges and meristic variation. J. Morphol..

[70] Johanson Z., Sutija M., Joss J. (2005). Regionalization of axial skeleton in the lungfish *Neoceratodus forsteri* (Dipnoi). J. Exp. Zool. Part B Mol. Dev. Evol..

[71] Niemelä E., Lajunen M., Kuusela J., Haantie J., Aro P., Kalske T. (2013). Scale reading atlas for Atlantic salmon in the Barents Sea area. Trilateral Cooperation on Our Common Resource, Proceedings of the the Atlantic Salmon the Barents Region—Kolarctic Salmon (KO197), Vadsø, Norway, 15 March 2011.

[72] Presnell J.K., Schreibman M.P. (1998). Humason’s Animal Tissue Techniques.

[73] Fjelldal P.G., Nordgarden U., Berg A., Grotmol S., Totland G.K., Wargelius A., Hansen T. (2005). Vertebrae of the trunk and tail display different growth rates in response to photoperiod in Atlantic salmon *Salmo salar* L., post-smolts. Aquaculture.

[74] Hamilton S.J., Mehrle P.M., Mayer F.L., Jones J.R. (1981). Method to evaluate mechanical properties of bone in fish. Trans. Am. Fish. Soc..

[75] Turner C.H. (2006). Bone strength: Current concepts. Ann. N. Y. Acad. Sci..

[76] Fjelldal P.G., Grotmol S., Kryvi H., Gjerdet N.R., Taranger G.L., Hansen T., Porter M.J.R., Totland G.K. (2004). Pinealectomy induces malformation of the spine and reduces the mechanical strength of the vertebrae in Atlantic salmon, *Salmo salar*. J. Pineal Res..

[77] Vera L.M., Lock E.-J., Hamre K., Migaud H., Leeming D., Tocher D.R., Taylor J.F. (2019). Enhanced micronutrient supplementation in low marine diets reduced vertebral malformation in diploid and triploid Atlantic salmon (*Salmo salar*) parr, and increased vertebral expression of bone biomarker genes in diploids. Comp. Biochem. Physiol. Part B Biochem. Mol. Biol..

[78] Pfaffl M.W. (2001). A new mathematical model for relative quantification in real-time RT-PCR. Nucleic Acids Res..

[79] Anderson K.C., Elizur A. (2012). Hepatic reference gene selection in adult and juvenile female Atlantic salmon at normal and elevated temperatures. BMC Res. Notes.

[80] Braden L.M., Barker D.E., Koop B.F., Jones S.R.M. (2012). Comparative defense-associated responses in salmon skin elicited by the ectoparasite *Lepeophtheirus salmonis*. Comp. Biochem. Physiol. Part D Genom. Proteom..

[81] Xie F., Xiao P., Chen D., Xu L., Zhang B. (2012). miRDeepFinder: A miRNA analysis tool for deep sequencing of plant small RNAs. Plant Mol. Biol..

[82] Gistelinck C., Gioia R., Gagliardi A., Tonelli F., Marchese L., Bianchi L., Landi C., Bini L., Huysseune A., Witten P.E. (2016). Zebrafish collagen type I: Molecular and biochemical characterization of the major structural protein in bone and skin. Sci. Rep..

[83] Zoch M.L., Clemens T.L., Riddle R.C. (2016). New insights into the biology of osteocalcin. Bone.

[84] Koyama Y., Rittling S.R., Tsuji K., Hino K., Salincarnboriboon R., Yano T., Taketani Y., Nifuji A., Denhardt D.T., Noda M. (2006). Osteopontin deficiency suppresses high phosphate load-induced bone loss via specific modulation of osteoclasts. Endocrinology.

[85] Yuan Q., Jiang Y., Zhao X., Sato T., Densmore M., Schüler C., Erben R.G., McKee M.D., Lanske B. (2014). Increased Osteopontin Contributes to Inhibition of Bone Mineralization in FGF23-Deficient Mice. J. Bone Miner. Res..

[86] Huitema L.F.A., Apschner A., Logister I., Spoorendonk K.M., Bussmann J., Hammond C.L., Schulte-Merker S. (2012). Entpd5 is essential for skeletal mineralization and regulates phosphate homeostasis in zebrafish. Proc. Natl. Acad. Sci. USA.

[87] Rutsch F., Ruf N., Vaingankar S., Toliat M.R., Suk A., Höhne W., Schauer G., Lehmann M., Roscioli T., Schnabel D. (2003). Mutations in ENPP1 are associated with “idiopathic” infantile arterial calcification. Nat. Genet..

[88] Mulero J.J., Yeung G., Nelken S.T., Ford J.E. (1999). CD39-L4 Is a secreted human apyrase, specific for the hydrolysis of nucleoside diphosphates *. J. Biol. Chem..

[89] Martin A., David V., Quarles L.D. (2012). Regulation and function of the FGF23/klotho endocrine pathways. Physiol. Rev..

[90] Baeverfjord G., Prabhu P.A.J., Fjelldal P.G., Albrektsen S., Hatlen B., Lundebye A. (2018). Mineral nutrition and bone health in salmonids. Rev. Aquac..

[91] Sambraus F., Hansen T., Daae B.S., Thorsen A., Sandvik R., Stien L.H., Fraser T.W.K., Fjelldal P.G. (2020). Triploid Atlantic salmon *Salmo salar* have a higher dietary phosphorus requirement for bone mineralization during early development. J. Fish Biol..

[92] Cotti S., Huysseune A., Larionova D., Koppe W., Forlino A., Witten P.E. (2022). Compression fractures and partial phenotype rescue with a low phosphorus diet in the Chihuahua zebrafish osteogenesis imperfecta model. Front. Endocrinol..

[93] Heuer R.M., Grosell M. (2014). Physiological impacts of elevated carbon dioxide and ocean acidification on fish. Am. J. Physiol. Integr. Comp. Physiol..

[94] Avila E.M., Tu H., Basantes S., Ferraris R.P. (2000). Dietary phosphorus regulates intestinal transport and plasma concentrations of phosphate in rainbow trout. J. Comp. Physiol. B.

[95] Sugiura S.H., Ferraris R.P. (2004). Contributions of different NaPi cotransporter isoforms to dietary regulation of P transport in the pyloric caeca and intestine of rainbow trout. J. Exp. Biol..

[96] Bird N.C., Mabee P.M. (2003). Developmental morphology of the axial skeleton of the zebrafish, *Danio rerio* (Ostariophysi: Cyprinidae). Dev. Dyn..

[97] Boivin G., Meunier P.J. (2002). The Degree of mineralization of bone tissue measured by computerized quantitative contact microradiography. Calcif. Tissue Int..

[98] Cubbage C.C., Mabee P.M. (1996). Development of the cranium and paired fins in the zebrafish *Danio rerio* (Ostariophysi, Cyprinidae). J. Morphol..

[99] Totland G.K., Fjelldal P.G., Kryvi H., Løkka G., Wargelius A., Sagstad A., Hansen T., Grotmol S. (2011). Sustained swimming increases the mineral content and osteocyte density of salmon vertebral bone. J. Anat..

[100] Solstorm F., Solstorm D., Oppedal F., Gunnar P. (2016). The vertebral column and exercise in Atlantic salmon—Regional effects. Aquaculture.

[101] Ytteborg E., Torgersen J.S., Pedersen M.E., Helland S.J., Grisdale-Helland B., Takle H. (2013). Exercise induced mechano-sensing and substance P mediated bone modeling in Atlantic salmon. Bone.

[102] Stagi S., Cavalli L., Iurato C., Seminara S., Brandi M.L., de Martino M. (2013). Bone metabolism in children and adolescents: Main characteristics of the determinants of peak bone mass. Clin. Cases Miner. Bone Metab..

[103] Sugiura S.H., Dong P.M., Hardy R.W. (2000). Primary responses of rainbow trout to dietary phosphorus concentrations. Aquac. Nutr..

[104] Heraud C., Hirschinger T., Baranek E., Larroquet L., Surget A., Sandres F., Lanuque A., Terrier F., Roy J. (2022). Detection and modulation of olfactory sensing receptors in carnivorous rainbow trout (*Oncorhynchus mykiss*) fed from first feeding with plant-based diet. Int. J. Mol. Sci..

[105] Porteus C.S., Hubbard P.C., Uren Webster T.M., van Aerle R., Canário A.V.M., Santos E., Wilson R.W. (2018). Near-future carbon dioxide levels impair the olfactory system of a marine fish. Nat. Clim. Chang..

[106] Porteus C.S., Roggatz C.C., Velez Z., Hardege J.D., Hubbard P.C. (2021). Acidification can directly affect olfaction in marine organisms. J. Exp. Biol..

[107] Velez Z., Roggatz C.C., Benoit D.M., Hardege J.D., Hubbard P.C. (2019). Short- and medium-term exposure to ccean acidification reduces olfactory sensitivity in gilthead seabream. Front. Physiol..

[108] Graff I.E., Waagbø R., Fivelstad S., Vermeer C., Lie Ø., Lundebye A.K. (2002). A multivariate study on the effects of dietary vitamin K, vitamin D3 and calcium, and dissolved carbon dioxide on growth, bone minerals, vitamin status and health performance in smolting Atlantic salmon *Salmo salar* L.. J. Fish Dis..

[109] Hannan K.D., Rummer J.L. (2018). Aquatic acidification: A mechanism underpinning maintained oxygen transport and performance in fish experiencing elevated carbon dioxide conditions. J. Exp. Biol..

[110] Ishimatsu A., Hayashi M., Lee K.-S., Kikkawa T., Kita J. (2005). Physiological effects on fishes in a high-CO_2_ world. J. Geophys. Res. Ocean..

[111] Khan J.R., Johansen D., Skov P.V. (2018). The effects of acute and long-term exposure to CO_2_ on the respiratory physiology and production performance of Atlantic salmon (*Salmo salar*) in freshwater. Aquaculture.

[112] Goss G.G., Laurent P., Perry S.F. (1994). Gill morphology during hypercapnia in brown bullhead (*Ictalurus nebulosus*): Role of chloride cells and pavement cells in acid-base regulation. J. Fish Biol..

[113] Weston C.W., Wen J.W. (1982). Purified Monoammonium Phosphate Process. U.S. Patent.

[114] Andersen F., Magge A., Julshamn K. (1996). An estimation of dietary iron requirement of Atlantic salmon, *Salmo salar* L.; parr. Aquac. Nutr..

[115] Tsay J., Yang Z., Ross F.P., Cunningham-Rundles S., Lin H., Coleman R., Mayer-Kuckuk P., Doty S.B., Grady R.W., Giardina P.J. (2010). Bone loss caused by iron overload in a murine model: Importance of oxidative stress. Blood.

[116] Zhang W., Xu J., Qiu J., Xing C., Li X., Leng B., Su Y., Lin J., Lin J., Mey X. (2018). Novel and rapid osteoporosismodel established in zebrafish using high iron stress. Biochem. Biophys. Res. Commun..

[117] Green J.A., Brannon E.L., Hardy R.W. (2002). Effects of dietary phosphorus and lipid levels on utilization and excretion of phosphorus and nitrogen by rainbow trout (*Oncorhynchus mykiss*). 2. Production-scale study. Aquac. Nutr..

[118] Green J.A., Hardy R.W., Brannon E.L. (2002). Effects of dietary phosphorus and lipid levels on utilization and excretion of phosphorus and nitrogen by rainbow trout (*Oncorhynchus mykiss*). 1. Laboratory-scale study. Aquac. Nutr..

[119] Ketola H., Westers H., Houghton W., Pecor C. (1991). Effect of diet on growth and survival of coho salmon and on phosphorus discharges from a fish hatchery. Am. Fish. Soc. Symp..

[120] Ketola H.G., Harland B.F. (1993). Influence of phosphorus in rainbow trout diets on phosphorus discharges in effluent water. Trans. Am. Fish. Soc..

[121] Rodehutscord M., Gregus Z., Pfeffer E. (2000). Effect of phosphorus intake on faecal and non-faecal phosphorus excretion in rainbow trout (*Oncorhynchus mykiss*) and the consequences for comparative phosphorus availability studies. Aquaculture.

[122] Vielma J., Lall S.P. (1998). Phosphorus utilization by Atlantic salmon (*Salmo salar*) reared in freshwater is not influenced by higher dietary calcium intake. Aquaculture.

[123] Wiesmann D., Scheid H., Pfeffer E. (1988). Water pollution with phosphorus of dietary origin by intensively fed rainbow trout (*Salmo gairdneri* Rich.). Aquaculture.

